# Mechanical stress combines with planar polarised patterning during metaphase to orient embryonic epithelial cell divisions

**DOI:** 10.1242/dev.202862

**Published:** 2024-05-17

**Authors:** Guy B. Blanchard, Elena Scarpa, Leila Muresan, Bénédicte Sanson

**Affiliations:** ^1^ Department of Physiology, Development and Neuroscience, University of Cambridge, Downing Street, Cambridge CB2 3DY, UK; ^2^ Cambridge Advanced Imaging Centre, University of Cambridge, Downing Street, Cambridge CB2 3DY, UK

**Keywords:** Morphogenesis, Cell division orientation, Mechanics, Stress anisotropy, Neighbour compression, Steric hindrance, Planar polarity, Myosin-II, Axis extension, *Drosophila*

## Abstract

The planar orientation of cell division (OCD) is important for epithelial morphogenesis and homeostasis. Here, we ask how mechanics and antero-posterior (AP) patterning combine to influence the first divisions after gastrulation in the *Drosophila* embryonic epithelium. We analyse hundreds of cell divisions and show that stress anisotropy, notably from compressive forces, can reorient division directly in metaphase. Stress anisotropy influences the OCD by imposing metaphase cell elongation, despite mitotic rounding, and overrides interphase cell elongation. In strongly elongated cells, the mitotic spindle adapts its length to, and hence its orientation is constrained by, the cell long axis. Alongside mechanical cues, we find a tissue-wide bias of the mitotic spindle orientation towards AP-patterned planar polarised Myosin-II. This spindle bias is lost in an AP-patterning mutant. Thus, a patterning-induced mitotic spindle orientation bias overrides mechanical cues in mildly elongated cells, whereas in strongly elongated cells the spindle is constrained close to the high stress axis.

## Teaser

**Summary:** This work demonstrates that patterning and stress cues, both tensile and compressive, are integrated at metaphase to direct the spindle orientation and thus the orientation of cell division.

## INTRODUCTION

The orientation of cell division (OCD) in the plane of epithelia is important for tissue morphogenesis and homeostasis. The orientation of greatest tissue level stress has been shown to influence the orientation of the mitotic spindle and hence the OCD, leading to stress relaxation (Campinho et al., 2013; Fink et al., 2011; Mao et al., 2013; Wyatt et al., 2015). Similarly, uniaxial compression has been shown to orient the mitotic spindle perpendicular to the orientation of compression (Lazaro-Dieguez et al., 2015; Lisica et al., 2022). Both anisotropic stress and compression result in cell elongation along or close to the axis of greatest relative stress (Nestor-Bergmann et al., 2019) providing a cell shape cue along which the mitotic spindle can orient, though direct sensing of stress anisotropy has been shown in some examples (Hart et al., 2017; Minc et al., 2011).

During developmental morphogenesis, patterning can also contribute to orienting cell divisions. In vertebrate embryos, divisions can be oriented by planar cell polarity (PCP) during axis elongation (Gong et al., 2004; Quesada-Hernandez et al., 2010). In *Drosophila* embryos, also during axis elongation, antero-posterior (AP) patterning has been shown to orient divisions along the AP axis in the posterior-most abdominal region (da Silva and Vincent, 2007). Later in *Drosophila* embryo development, cells abutting parasegment boundaries (PSBs) on average divide along the AP axis because of Myosin-II enrichment along the PSB, which restricts spindle pole movement (Scarpa et al., 2018). However, the majority of cells in this embryonic epithelium divide along the dorso-ventral (DV) axis, as predicted by their interphase long axis of cell shape (Scarpa et al., 2018), suggesting that a mixture of patterning and mechanics might be involved.

A large body of work supports the Hertwig rule that the interphase long axis of a cell predicts its OCD (Gibson et al., 2011; Gray et al., 2004; Hertwig, 1884; Minc et al., 2011; Wyatt et al., 2015). Because most cells round up during mitosis both in culture (Kunda et al., 2008) and *in vivo* (Chanet et al., 2017) to provide enough room to accommodate mitotic spindle assembly and rotation, the Hertwig rule implies that a molecular mechanism must exist for the mitotic cell to ‘remember’ the interphase cell elongation orientation through mitosis. Such a mechanism has been identified in the *Drosophila* notum involving the interphase accumulation of Mud (NuMa in vertebrates), which binds to astral microtubules during metaphase. In this epithelium, Mud is located at tri-cellular junctions, providing a memory of interphase topology through mitosis (Bosveld et al., 2016).

However, in other fly epithelia such as the follicular and embryonic epithelia, Mud is not found at tricellular junctions and divisions do not always align with interphase cell shape (Finegan et al., 2019; Scarpa et al., 2018; Wang et al., 2017). In the planar polarised fly embryonic neurectoderm, the distribution of planar OCDs is not changed in mutants of Mud or mutants or RNAi of its binding partner Pins (LGN/GPSM2 in vertebrates) (Scarpa et al., 2018). In other domains of the embryonic epithelium, Pins polarisation aligns with stress patterns, with the loss of Pins polarisation randomising the OCD (Camuglia et al., 2022). In these and other contexts, the spindle can be re-oriented in metaphase by mechanical cues (Camuglia et al., 2022; Lazaro-Dieguez et al., 2015; Scarpa et al., 2018) or Myosin-II perturbation (Scarpa et al., 2018), overriding or replacing interphase cues. Thus, how mechanical and patterning cues persist from interphase through mitosis and, if in conflict, how these cues achieve a compromise OCD is unclear.

In this study, we focus on cell cycle 14 in *Drosophila* embryos, which has a long interphase starting at cellularisation, with the first mitoses occurring during gastrulation (Foe, 1989). During *Drosophila* axis extension, AP-patterning leads to intrinsic DV-oriented contractility driven by planar polarised Myosin-II at cell–cell junctions (Rauzi et al., 2008; Tetley et al., 2016; Zallen and Wieschaus, 2004). The early embryonic epithelium is thus planar polarised when cell cycle 14 mitoses start, with Myosin-II polarity a clear readout of this polarisation (Sharrock and Sanson, 2020; Tetley et al., 2016). To ask how stress and patterning combine *in vivo* to orient divisions in the plane of the epithelium, we analysed quantitatively hundreds of epithelial cell divisions, characterising the contribution to OCD of stress anisotropy, cell elongation and Myosin-II polarisation cues. This work demonstrates that patterning and stress cues, both tensile and compressive, are integrated at metaphase to direct the spindle orientation.

## RESULTS

### Using semi-automated cell tracking to follow the time course of epithelial cell divisions *in vivo*

**Fig. 1.**
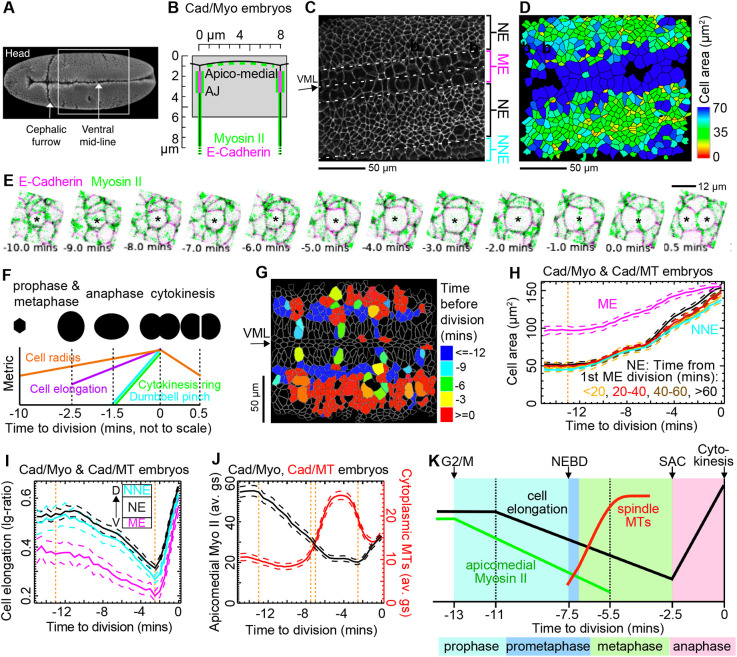
**Tracking cell divisions *in vivo*.** (A) Ventral *Drosophila melanogaster* embryo view showing approximate imaged trunk/abdominal region (boxed area). (B) Schematic of epithelial cell apex showing the depth projection range (grey box) relative to cell apices used to track cells at the level of adherens junctions (AJs) in Cad/Myo movies. (C) Projection of E-Cadherin confocal channel at the level of AJs at 0 min from movie CS_3. Central off-horizontal band of large cells are mesectoderm (ME) cells, straddling the ventral midline (VML). Neurectoderm (NE) cells flank the ME, with some rounding up non-neural ectoderm (NNE) cells in view laterally. Anterior is left. (D) Segmented cell shapes of well-tracked cells in C, colour-coded by surface area. (E) Example dividing cell (marked with asterisk) from a Cad/Myo (magenta/green, respectively) embryo (see Movie 2), from −10 min before to +30 s after cytokinesis. The cell view has been rotated so that orientation of division is horizontal. (F) The gradients of five metrics are used to classify cytokinesis events (Fig. S1F-H; Materials and Methods). (G) Still from Movie 1 (bottom right panel) at 49 min, showing cells from embryo CS_4 classified as dividing, colour-coded by time before division. Cells in red have divided, whereas blue cells will divide within the next 50 min. Anterior is left. (H) Cell area at the level of AJs through mitosis for five Cad/Myo and four Cad/MT embryos. NE (total *N*=505) cells are separated into four developmental time bins (*N*=27, 189, 169, 120, respectively). NNE (*N*=178) and larger ME (*N*=81) cells all follow the same time course of area increase. (I) Cell elongation at the level of AJs through mitosis for five Cad/Myo and four Cad/MT embryos. Log-ratio is log(long axis length/short axis length) from best-fit ellipses. ME cells are more isotropic than NE and NNE cells but follow the same time course (*N* as in H). (J) Fluorescence patterns through mitosis. Left *y*-axis, apicomedial Myosin-II (sqh-mCherry) density for Cad/Myo embryos (black line). Right *y*-axis, cytoplasmic MT (Jupiter-mCherry) density for Cad/MT embryos (magenta line). (K) Schematic summary of the time course of *Drosophila* round 14 divisions. G2/M, effective G2 to mitosis transition; NEBD, nuclear envelope breakdown; SAC, spindle assembly checkpoint marking anaphase onset. Black dotted lines mark the onset of cell elongation and the trough of apicomedial Myosin-II density. In H-J, dashed lines either side of pooled population means are ±95% CI. Orange vertical dotted lines show the effective onset of mitosis at −13 min, NEBD at −7.5 min, start of metaphase at −7 min and start of anaphase at −2.5 min.

We set out to analyse the cell divisions in a model epithelium, the thoracic/abdominal ectoderm in the early *Drosophila melanogaster* embryo. In this embryo, nuclei go through 13 cycles of synchronous divisions before cellularisation, controlled by maternal transcripts (Tadros and Lipshitz, 2009). After cellularisation, cycle 14-16 divisions are only locally synchronous and controlled by the zygotic expression of *string*/*cdc25* (Edgar and O'Farrell, 1990; Foe, 1989). To follow cycle 14 epithelial cell divisions in real time, we live imaged the ventral side of the embryo in the thoracic/abdominal region (Fig. 1A) for up to 2 h at 21°C. We acquired two types of dataset, five movies of *sqh^AX3^; (endo)DE-Cadherin-GFP, sqh-mCherry* embryos at 30 s frame intervals for E-Cadherin and Myosin-II quantification, which we will call Cad/Myo, and four movies of *(ubi)DE-Cadherin-GFP; jupiter-mCherry* embryos, at 20 s frame intervals for quantifying E-Cadherin and microtubules (MTs), which we will call Cad/MT (see Materials and Methods). Cell contours at the level of adherens junctions (AJs) using the E-Cadherin signal (Fig. 1B-D; Fig. S1A-C) were segmented and tracked through time as previously described (Blanchard et al., 2009; Butler et al., 2009; Tetley et al., 2016; see Materials and Methods).

In our ventral view movies, two lines of mesectoderm (ME) cells either side of the ventral midline (VML) are always in view, flanked on either side by a larger neurectoderm (NE) domain (Fig. 1C; Fig. S1B). Beyond the NE, non-neural ectoderm (NNE) cells are in view or come into view as the NE converges towards the VML or the embryo rolls. The NNE is located between the NE and amnioserosa (outside of the field of view). The NNE and ME are identifiable as the first domains to divide in our movies. All movies were synchronised to the timepoint at which the first ME cell in the field of view reached cytokinesis (e.g. frame 3 in Movie 1). This was used as developmental time zero for this study, which corresponds to ∼35 min after the start of axis extension (Tetley et al., 2016). Together, the five Cad/Myo movies span a period from −20 min to 100 min (Fig. S1D) covering stages 8 to 10 (Bate and Martinez Arias, 1993), with the four Cad/MT movies spanning −10 min to 65 min (Fig. S1E).

We next developed a classifier algorithm, using an approach similar to Wang et al. (2017), to identify cell divisions (Fig. 1E) within the population of tracked cells, using gradients over time of cell shape and fluorescence metrics (Fig. 1F; Fig. S1F-H; Movies 1 and 2; see Materials and Methods). More than 90% of dividing cells in three manually curated Cad/Myo movies and more than 50% in two non-manually curated Cad/Myo movies were correctly identified. The overall false positive rate for Cad/Myo and Cad/MT movies was 0.5%, which were removed from all analyses. In total, we correctly tracked 764 divisions in nine movies (Fig. S1I,J) with a minimum tracking time of 10 min for each cell before cytokinesis. We synchronised each cell division time course relative to the last movie frame before a new E-Cadherin junction formed between new daughter cells, defining a time zero at cytokinesis for all cell divisions (penultimate panel in Fig. 1E,G; Movie 1). We also assigned a division axis to each dividing cell, corresponding to the orientation of daughter centroids in the first frame after division (Fig. 1E; Movie 2), to which we could then compare the orientation of other cues before cytokinesis.

To compare cell divisions, we first asked whether there are differences in the rate of progress through mitosis between domains, such as the start of rounding, appearance of the mitotic spindle and cytokinesis ring. We found that the evolution of average NE cell shapes leading up to cytokinesis was remarkably similar across developmental time (Fig. 1H). As cells round up upon mitotic entry, we observed a cell area increase in the plane of the AJs first detectable at −12 min, continuing through to cytokinesis. ME cells have larger apices than the more columnar NE cells, but they follow a remarkably similar time course of area increase (Fig. 1H). Cell elongation decreased from −11 min until −2.5 min, and then increased with anaphase elongation (Fig. 1I). Apicomedial Myosin-II (Fig. 1B) density started to reduce at −13 min (Fig. 1J), which is the earliest detectable change we found associated with cell division. Cytoplasmic MT fluorescence density (Fig. S1K,L) increased with the formation of the mitotic spindle, peaking at −3 min just before the start of anaphase (Fig. 1J).

For a visual confirmation of mitotic timings, we generated a representation of average cell fluorescence through division (see Materials and Methods). We overlaid and averaged fluorescence intensities surrounding the cell centroids (Fig. S1M) of all divisions, rotating local image data so that each division orientation was horizontal. We did this at the level of AJs and 4 µm deeper at a sub-AJ level for Cad/Myo (Fig. S1N; Movie 3) and Cad/MT (Fig. S1O; Movie 4) movies. In the MT (Jupiter-mCherry) channel, the low fluorescence signal of MTs at −7 min 40 s was from centrosomes at the base of the nucleus immediately before nuclear envelope breakdown (NEBD). The brighter mitotic spindle was then variably oriented within and out of the plane at −5 min 40 s, settling in the plane along the future OCD at −3 min. The cytokinesis ring appeared at −2.5 min, marking the start of anaphase.

Taking advantage of this fine-grained characterisation of cell shapes, MT and Myosin-II dynamics (Fig. 1K), we defined the initial decrease of apicomedial Myosin-II at −13 min as our effective G2/M transition. Prometaphase started at −7.5 min with NEBD, followed immediately by the formation of the metaphase spindle. Metaphase then spanned −7 min to −3 min, before anaphase elongation commenced. This mitotic temporal schedule was remarkably consistent across cells in different domains. We next focused on the orientation of divisions.

### Tissue tension and compression lead to cell elongation which correlates with the OCD

Cycle 14 cell divisions in the embryonic epithelium occur in locally synchronised mitotic domains which have been identified previously in fixed embryo samples (Foe, 1989; Hartenstein et al., 1994), but have not yet been comprehensively characterised in live embryos. From imaging of specific tissues, the OCD is known to be biased to DV in the NE (Scarpa et al., 2018; Sharrock and Sanson, 2020) but to AP in the ME (Camuglia et al., 2022; Wang et al., 2017). We therefore set out to assign all cell divisions we identified in real time to known mitotic domains before assessing patterns of OCD.

**Fig. 2.**
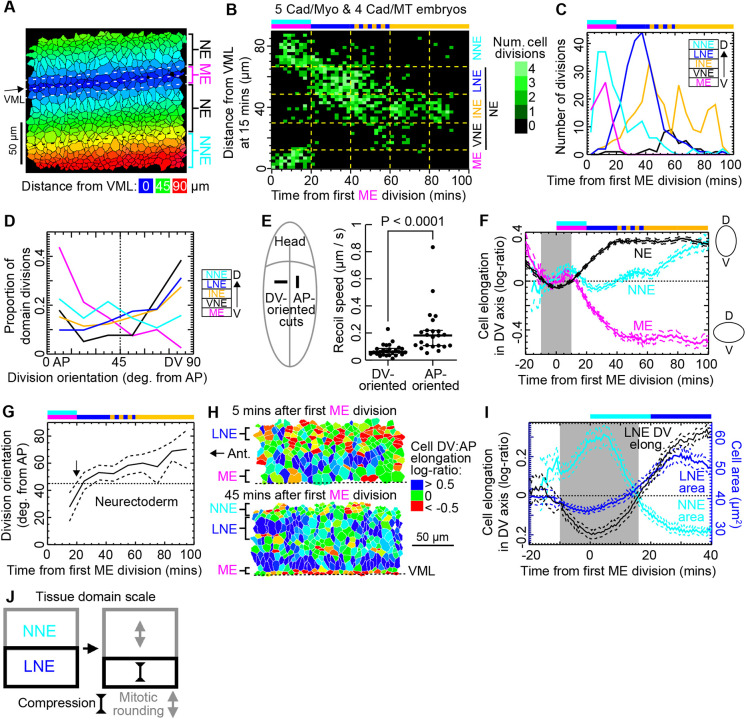
**Metaphase cell shape re-orients over developmental time.** (A) DV co-moving coordinates [distance from the ventral midline (VML) in µm] were assigned to cells at 15 min after the first mesectoderm (ME) division, here for embryo CS_2. Blue ME cells (DV<12 µm) are flanked by cyan-green neurectoderm (NE, DV=12-66 µm), with lateral orange-red non-neural ectoderm (NNE, DV>66 µm). (B) Frequency of cell division events from five Cad/Myo and four Cad/MT embryos, broken down by DV co-moving coordinate and 20-min development time bins (yellow dashed grid). VNE, INE and LNE are ventral, intermediate and lateral NE, respectively. Top bar shows which domains dominate divisions in each 20-min bin, colours as in DV domain legend on right. All *N*=764 are cycle 14 divisions, no cell divided twice. (C) Frequency of cell divisions identified over time in Cad/Myo and Cad/MT embryos in the five spatio-temporal domains identified in B. *N*: ME=81, VNE=38, INE=235, LNE=232, NNE=178. Bin size is 5 min. Top bar as in B. (D) Frequency of division orientation broken down by mitotic domain, expressed as a proportion of total domain *N*. *N* per domain same as in C. Bin size is 15°. (E) Initial retraction speeds after tissue-scale laser ablation (unpaired two-tailed Mann-Whitney test; *N*=25 each). Schematic on left shows the location of AP- and DV-oriented tissue scale cuts in the ventral extended germband**.** (F) Cell elongation log-ratio in the DV axis for ME, NNE and pooled NE domains of five Cad/Myo and four Cad/MT embryos over developmental time. Grey highlights epoch when NE cell orientation is on average towards AP. Top bar as in B. See Fig. S2E,F for *N* and cell areas. (G) OCD over developmental time for NE cells in five Cad/Myo and four Cad/MT embryos. Arrow at 20 min shows transition from AP- to DV-oriented divisions. Top bar as in B. Total *N*=505. (H) Images showing tracked cell shapes in the LNE switching from AP-oriented (red) at 5 min (top) to DV-oriented (blue) at 45 min (bottom). One side of embryo CS_4 is shown from the VML (bottom) to the NNE (top). Anterior is left. (I) Focus on cells shapes in the LNE and adjacent NNE domains before and during LNE divisions. Cells in the NNE domain (cyan line) divide at 0-20 min, enlarging the domain, whereas LNE cells temporarily become AP-elongated (black line, grey highlight) and reduce area (blue line). Horizontal bars for the NNE and LNE divisions as in B. For *N* see Fig. S2E. (J) Schematic of proposed apical expansion of the dividing NNE domain that temporarily compresses the LNE in DV. Dashed lines either side of population means in F,G,I are ±95% CIs.

We first set up a DV coordinate axis. When the first divisions start in our movies, the embryonic epithelium is still undergoing axis extension, converging along its DV axis until 15 min after the first ME division (Fig. S2A). After this time, the lateral border of the NE, distinguishable from the dorsally adjacent NNE cells which divide early along with ME cells, is reliably close to 66 µm from the VML across movies (Fig. S2A). The DV ‘identity’ of each cell was therefore set to its DV coordinate at 15 min (Fig. 2A; Movie 5).

We used this quantitative mapping to assign cells to one of five DV regions that had distinct patterns of cell area over time (Fig. S2B,C). These patterns reflected cell division timings and were consistently at the same DV locations across five Cad/Myo and four Cad/MT movies (Fig. 2B). ME cells (less than 12 µm from VML) divide at the same time (0-20 min) as the most lateral NNE domain (>66 µm from VML). Between these, we separated the NE into ventral (VNE), intermediate (INE) and lateral (LNE) neurectoderm. These three domains correspond approximately to the expression domains of the three homeodomain transcription factors Vnd (Ventral nervous system defective), Ind (Intermediate neuroblast defective) and Msh (Muscle segment homeobox; also known as Dr) which pattern the NE along DV (see Materials and Methods) (Cornell and Von Ohlen, 2000; Reeves and Stathopoulos, 2009). Within the NE, the LNE domain (48-66 µm, 20-50 min) divides first, overlapping with the start of the INE domain (30-48 µm, 40-100 min). Most VNE cells (12-30 µm) divide after 100 min, beyond the developmental time window analysed here. The temporal sequence of divisions in the five DV domains we identified in live embryos (Fig. 2C) agreed with the temporal sequence of equivalent domains previously identified from fixed samples (Foe, 1989).

We next focused on how division orientation is distributed in the different mitotic domains. We confirmed that ME cell divisions are preferentially oriented along the AP embryonic axis, whereas divisions in the three NE domains, though variable, are biased towards DV (Fig. 2D). NNE cells are mildly AP-biased.

We hypothesised that NE cells are DV-biased because of greater tissue tension in DV. Though tissue-scale laser ablation experiments during the fast phase of axis extension have shown that tissue tension in the embryonic epithelium is greater in DV than AP (Collinet et al., 2015), tissue tension has not been probed during the slow phase of axis extension and beyond, when cycle 14 divisions occur. We therefore performed tissue-scale laser cuts on the apical myosin meshwork in perpendicular orientations at 25-30 min, immediately after the first NE divisions (Fig. 2B). We found that tissue stress anisotropy is indeed strongly DV-oriented (Fig. 2E; Fig. S2D; Materials and Methods).

A second prediction of greater DV tissue tension is that cells should be on average DV-elongated. Indeed, NE cell orientations were strongly DV-biased from 25 min onwards (Fig. 2F; Fig. S2E,F), accounting for the majority of NE divisions (Fig. 2B,C). Thus, the DV bias we observed in NE division orientation suggested that anisotropic tissue stress stretches cells in DV.

Unexpectedly however, between −10 and +10 min (grey highlight in Fig. 2F), the NE cells transiently lost their DV-oriented elongation, instead becoming AP-oriented. We hypothesised that this early AP cell elongation should lead to AP-oriented divisions. Indeed, NE divisions before 20 min were AP-oriented (Fig. 2G). This correlated switch in cell orientation and OCD could be due to changes in stress patterns in the NE. This change in local stress could be caused by synchronised cell divisions occurring in adjacent tissues, as dividing epithelial cells round up by pulling up their bases, leading to apical expansion. As in other tissues (Gupta et al., 2021) this is likely to compress nearby cells. Our temporal mapping of cell divisions showed that the earliest NE divisions are in the LNE, which is flanked dorsally by the NNE and which divides immediately before LNE divisions (Fig. 2B). We therefore hypothesised that apical expansion associated with mitotic rounding in this flanking domain might be compressing the LNE cells. We first noted that LNE cells were the most strongly AP-oriented NE cells during NNE divisions (Fig. 2H,I; Fig. S2G). Furthermore, there was a tight temporal coupling between the area expansion of the NNE during division and the area reduction, a signature of mechanical compression, and a switch to AP-oriented elongation in the LNE between −10 and 16 min (Fig. 2I, grey highlight). Thus, the patterns and timings of cell area and elongation in the NNE and LNE suggested that the dividing NNE cells compress the earliest mitotic cells in the LNE, re-orienting their divisions (Fig. 2J).

We show at the population level that cell elongation and the OCD are temporally correlated, suggesting that elongation changes (due to stress changes) through mitosis are affecting the OCD. To understand better how local changes in stresses affect cell division orientation, we next investigated on a by cell basis whether and when compression during mitosis can re-orient the OCD.

### Compression from neighbouring dividing domains and cells during metaphase re-orients divisions

**Fig. 3.**
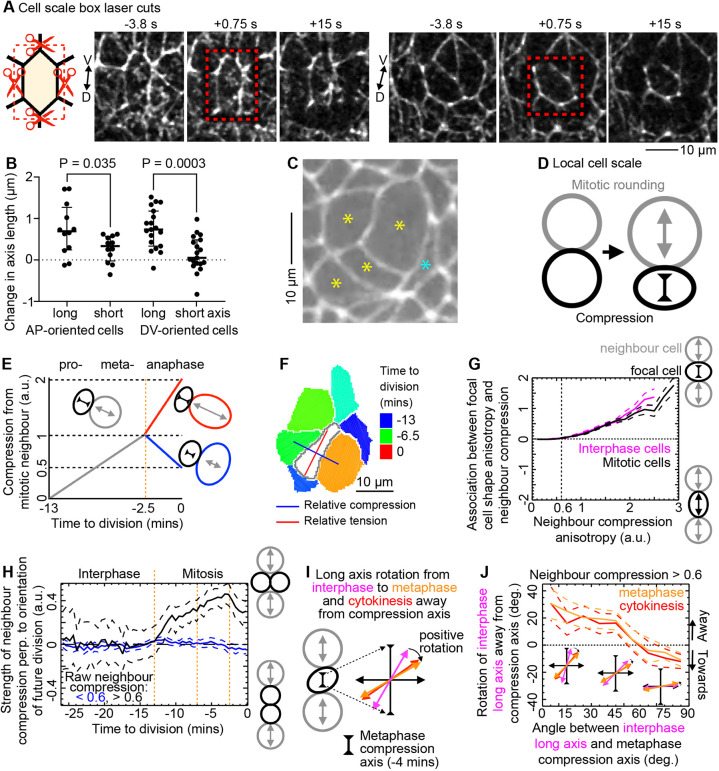
**Compression from neighbouring dividing cells during metaphase re-orients divisions.** (A) For box laser cut ablations, schematic depicts how a rectangular template is constructed to cut neighbour–neighbour junctions around the focal cell. Three stills from two example box laser ablations are shown, 4 s before, immediately after (with red box ablation template) and 15 s after the cut, showing the severed neighbour–neighbour junctions and retraction of the focal cell. The first example cell is elongated in DV, the second in AP, before the cut. (B) Reduction in axis lengths after box ablation broken down by orientation of cell long axis before ablation (paired one-way Anova, Sidak multiple comparison test; AP-oriented, *N*=12; DV-oriented, *N*=19) (see Fig. S3A-D). (C) Example nest of quasi-synchronised divisions (yellow asterisks) and their compressive effect on each other and neighbouring cells (blue asterisk). (D) Cartoon of anisotropic compression by neighbouring cell divisions. (E) Schematic showing how time to division in a neighbouring cell (grey, red, blue) is converted to a strength of compression on the focal cell (black) (see Fig. S3E). Compression strength is normalised to 1 at the end of metaphase (orange line). In anaphase the relative orientation of the neighbouring dividing cell is accounted for, as the compression effect is greater if along the future division axis (red). (F) Example focal cell (white) surrounded by cells at various stages of mitosis. Large neighbours close to cytokinesis (orange and green) are on opposite sides, leading to a strongly anisotropic stress tensor (red and blue cross). (G) Neighbour compression anisotropy starts to re-shape focal interphase and mitotic cells above a threshold value of 0.6 (a.u.). See Fig. S3F for *N*. (H) Strength of neighbour compression in the future OCD from interphase through mitosis. Positive values indicate when neighbour compression predicts perpendicular OCD. Orange dotted lines mark the onset of mitosis (−13 min), metaphase (−7 min) and anaphase (−2.5 min). *N* divisions: <0.6, 128; >0.6, 279. (I) Scheme for how neighbour compression in metaphase should induce a rotation of the interphase cell long axis (magenta) away from the compression axis (here vertical) in metaphase (orange), thereby re-orienting the OCD (red). (J) Re-orientation of the interphase cell long axis in metaphase by neighbour compression re-sets the OCD. Some rotation of the cell long axes away from the compression orientation is expected when these are aligned (*x*-axis=0°) and some rotation towards the compression orientation is expected if these are already perpendicular (*x*-axis=90°) from undirected dynamics. In between these angle limits, the metaphase long axis (orange) and OCD (red) are rotated away from the interphase long axis and towards the compression axis, unlike in cells with weak neighbour compression (<0.6; Fig. S3G). Inset cartoon axes show approximate mean long axis rotations at *x*-axis angles of 15°, 45° and 75°. Total *N*=279 for divisions in all domains where anisotropic neighbour compression was strong (>0.6) in metaphase (−4 min). In G,H,J, data are pooled from five Cad/Myo and four Cad/MT embryos. Dashed lines straddling population means are ±95% CIs.

We have shown that the OCD is correlated with the orientation of cell long axes. As a test of the link between cell elongation and stress anisotropy, we asked whether the orientation of cell elongation is the high stress axis of the cell. Indeed, the cell long axis has been shown to be a reliable readout of the axis of greatest local stress in other tissues (Nestor-Bergmann et al., 2018, 2019). We tested this hypothesis in the embryonic epithelium by carrying out ‘box’ laser cuts to isolate the apical surface of interphase cells from their neighbours (Fig. 3A). We found that the orientation of largest recoil was systematically correlated with the cell long axis, irrespective of the orientation of the cell (Fig. 3B; Fig. S3A-D). Thus, cell elongation is associated with local anisotropic stress, greater in the cell long axis than short axis. This supports the hypothesis that there is a switch from tissue DV compression to DV tension at 15-20 min in the NE leading to a correlated switch in OCD.

We wondered whether a similar neighbour compression effect could be re-orienting divisions at a more local scale, within mitotic domains. Indeed, it remained a puzzle that the orientation of divisions in the NE, though DV-biased, was highly variable (Fig. 2D). Tissue stress anisotropy orients cells on average, but local stress can vary (Nestor-Bergmann et al., 2018). Nests of quasi-synchronised cell divisions are common in cycle 14 *Drosophila* divisions (Fig. 3C; Movies 1 and 6) (Foe, 1989), so we hypothesised that neighbouring divisions, as natural physical perturbations during mitosis (Mao and Baum, 2015), could re-orient the OCD and explain the broad distribution of OCD in the NE.

In epithelia, expanding mitotic cells may compress neighbouring cells (Fig. 3D) (Gupta et al., 2021). To estimate the anisotropy of compression of apically enlarging mitotic neighbours around each cell and its effect on the anisotropy of cell shape, we constructed a compression shear tensor for each cell in each movie frame (see Materials and Methods). For this, we assumed that the average enlargement of mitotic cell radii (Fig. S3E) linearly predicts the relative compression exerted on neighbours (Fig. 3E). The anisotropic compression tensor then summarises the compression of all neighbours, depending on their orientation and scaled by the length of their shared interface (example in Fig. 3F).

We first tested whether the strength of the neighbour compression tensors was related to the strength of focal cell elongation in the predicted orientation. Indeed, these were positively associated (using a composite measure of similarity of magnitude and orientation, see Materials and Methods) above a threshold compression strength of 0.6 (a.u.) for both interphase and mitotic cells (Fig. 3G; Fig. S3F). Above this threshold, neighbour compression predicts OCD only during mitosis, most strongly in metaphase (Fig. 3H), suggesting that neighbour compression in metaphase can re-orient the OCD.

To better understand mechanistically how neighbour compression re-orients OCD, we asked whether the interphase (−13 min) long axis is re-oriented by metaphase (−4 min) neighbour compression (Fig. 3I). We found that the cell long axis rotated towards the neighbour compression axis, by around 20° on average, depending on the angular difference between interphase long axis and neighbour compression axis, only for cells subject to a neighbour compression strength above 0.6 (a.u.) (Fig. 3J and Fig. S3G, orange lines). Furthermore, the subsequent division axis was set by this rotation (red lines).

We conclude that anisotropic compression from dividing neighbouring domains (previous section) and neighbouring cells (this section) forces a new cell long axis orientation and that, if this occurs during metaphase, this also sets the division axis.

### An AP-oriented cue attracts the mitotic spindle independently of mechanics

**Fig. 4.**
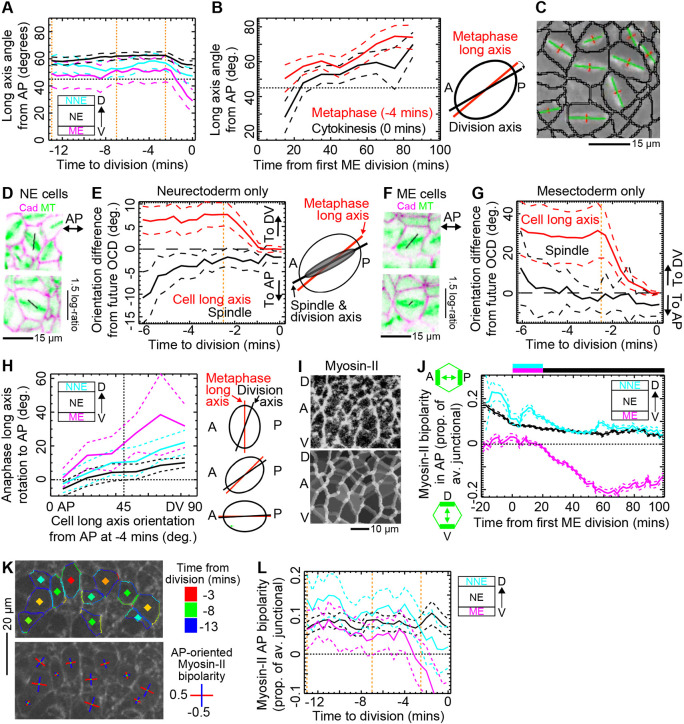
**An AP-oriented cue attracts the mitotic spindle independently of mechanics.** (A) The average orientation of cell long axes relative to the AP axis for cells in three ectoderm domains during mitosis. Cell long axes rotate to AP during anaphase, most strongly in the mesectoderm (ME). Data pooled from five Cad/Myo movies. *N* divisions: NNE, 73; NE, 336; ME, 40. (B) Metaphase cell long axis orientation compared with division axis at cytokinesis (OCD) across developmental time for neurectoderm (NE) divisions only (*N*=505). Data in A,B are pooled from five Cad/Myo and four Cad/MT movies. (C) Example segmented spindles (green lines, principal components; red lines, orthogonal components) taken from Movie 6. (D) Example NE cells in metaphase (−4 min) taken from Movie 7, showing the spindle towards AP (horizontal) of long axis of the best-fit ellipse to cell shape (black line). (E) Angular difference, to AP or DV, between both the cell long axis and spindle and the OCD for NE cells. The long axis of NE cells rotates ∼7° on average towards the spindle and the future OCD during anaphase, whereas the metaphase spindle orientation predicts the OCD. Data pooled from four Cad/MT embryo movies. *N* divisions=169. (F) Example ME cells as in D. (G) As E, but for ME divisions. Cell long axes rotate ∼30° on average towards the spindle and the future OCD during anaphase. *N* divisions=41. (H) The degree of long axis rotation to AP in anaphase depends on the initial metaphase long axis orientation from AP, with DV-oriented cells capable of, and displaying, the highest rotation. *N* divisions as in A. Data pooled from five Cad/Myo and four Cad/MT movies. N divisions: NNE, 178; NE, 505; ME, 81. (I) Myosin-II planar polarity. Top; example Cad/Myo embryo raw junctional Myosin-II fluorescence. Bottom; segmented junctions for same view, colour-coded by junctional Myosin-II density (greyscale). A, anterior; D, dorsal; V, ventral. (J) Mean Myosin-II planar polarity over developmental time by mitotic domain, projected onto the AP axis. Top bar as in Fig. 2B, but with LNE and INE domains merged to NE in black. Data pooled from five Cad/Myo embryos. For total number of well-tracked cells, see Fig. S1D. (K) Myosin-II planar polarity of example LNE cells during mitosis. Top and bottom are the same snapshot of the raw Myosin-II channel from a Cad/Myo embryo. Top: calculated junctional Myosin-II fluorescence intensity has been drawn on top of junctions (blue low, red high). Cell centroids are coloured by time before division. Bottom: the strength and orientation of planar polarity has been drawn on each mitotic cell. AP is horizontal. (L) Myosin-II planar polarity projected onto the AP axis for different mitotic domains during mitosis. Positive planar polarity is AP-oriented. Data pooled from five Cad/Myo embryos. *N* divisions: NNE, 73; NE, 336; ME, 40. In all graphical panels, dashed lines straddling population means are ±95% CIs. Vertical orange dotted lines in A,E,G,L mark the effective onsets of prophase (−13 min), metaphase (−7 min) and anaphase (−2.5 min).

In addition to mechanical cues, previous studies have shown that AP-patterning can influence division orientation in the *Drosophila* embryonic epithelium (da Silva and Vincent, 2007; Scarpa et al., 2018). To address how and when patterning might be influencing the OCD, we investigated how cell orientation relative to the AP axis changes throughout mitosis. Surprisingly, we found that there is a significant rotation of the cell long axis on average towards AP in all domains, but only during anaphase (Fig. 4A). This rotation could either be because cells have a memory of recent interphase orientation (e.g. the AP-oriented elongation of LNE cells during transient NNE compression discussed above) that they are returning to, or because there is a stress-independent bias to AP. Supporting the latter, across our developmental time period the division angle was consistently rotated to AP from the metaphase (−4 min) long axis orientations (Fig. 4B). This indicates that the anaphase rotation was not linked to the temporary early compression of the LNE and was therefore not due to an interphase memory of shape or stress.

To understand whether the mitotic spindle rotates with the long axis of cell shape or whether the long axis rotates to match the position of the mitotic spindle, we tracked mitotic spindles in 3D within each dividing cell, from their formation after NEBD (−7 min) until cytokinesis (Fig. 4C; Fig. S4A-C; Movie 6). Strikingly, at the end of metaphase (−2.5 min), the spindle was more closely aligned to the future OCD than to the cell long axis in NE cells (Fig. 4D,E). During anaphase, as cells elongate because of polar relaxation (Rodrigues et al., 2015), the long axis then rotated towards the spindle axis and towards AP. A similar but more dramatic rotation of the long axis to AP was seen in ME cells (Fig. 4F-H) and NNE (Fig. S4D).

What is the cue attracting spindles towards AP? Numerous proteins are planar polarised downstream of AP-patterning (Pare and Zallen, 2020) and could attract (or repel) the spindle. Among these, Myosin-II is strongly planar polarised to DV-oriented junctions during axis extension (Bertet et al., 2004; Rauzi et al., 2008; Tetley et al., 2016; Zallen and Wieschaus, 2004) and is an essential component of the actomyosin cortex that has an instructive role in spindle orientation (di Pietro et al., 2016). Indeed, Myosin-II is required for centrosome separation (Rosenblatt et al., 2004) and clustering (Rhys et al., 2018), for spindle orientation during oocyte meiosis (Bourdais et al., 2023). Moreover, we have previously shown that cells adjacent to the Myosin-II-enriched PSBs orient their divisions towards the PSB, along AP, even if they are mildly elongated in DV (Scarpa et al., 2018). We therefore asked whether Myosin-II planar polarity is correlated with the spindle rotating towards AP in Cad/Myo embryos, using the OCD as a proxy for metaphase spindle orientation (Fig. 4E,G; Fig. S4D) in the absence of an MT channel.

We calculated the orientation and strength of Myosin-II planar polarity and projected this onto the AP axis (Fig. S4E; Fig. 4I; Materials and Methods) (Tetley et al., 2016). Planar polarity was strongest during the fast phase of axis extension (−20 min to 0 min), as expected, but some planar polarisation remained during cycle 14 cell divisions (0 min to 100 min) (Fig. 4J). Myosin-II planar polarity was AP-oriented on average in all domains at the developmental time at which cells divide (horizontal bars in Fig. 4J). Interestingly, we also found that in mitotic cells, Myosin-II planar polarity was significantly AP-oriented in all mitotic domains through metaphase (Fig. 4K,L).

We conclude that Myosin-II planar polarity, downstream of AP-patterning, remains present in cycle 14 mitotic cells in the embryonic epithelium. As the mitotic spindle orientation is biased towards AP from cell long axes, we therefore next tested whether this spindle orientation bias remains when AP-patterning is abrogated.

### Anaphase long cell axis rotation towards AP is lost in an AP-patterning mutant

To test whether AP-patterning is responsible for reorienting the mitotic spindle towards AP, we imaged double mutant and sibling control *knirps, hunchback* embryos into which we had introduced *sqh, GAP43* fluorescent reporters (see Materials and Methods). Double mutant embryos (*knihb*) lack essential gap genes responsible for setting up AP-patterning in the trunk and so lack the intrinsic active cell intercalation forces required to drive axis extension (Butler et al., 2009; Irvine and Wieschaus, 1994).

**Fig. 5.**
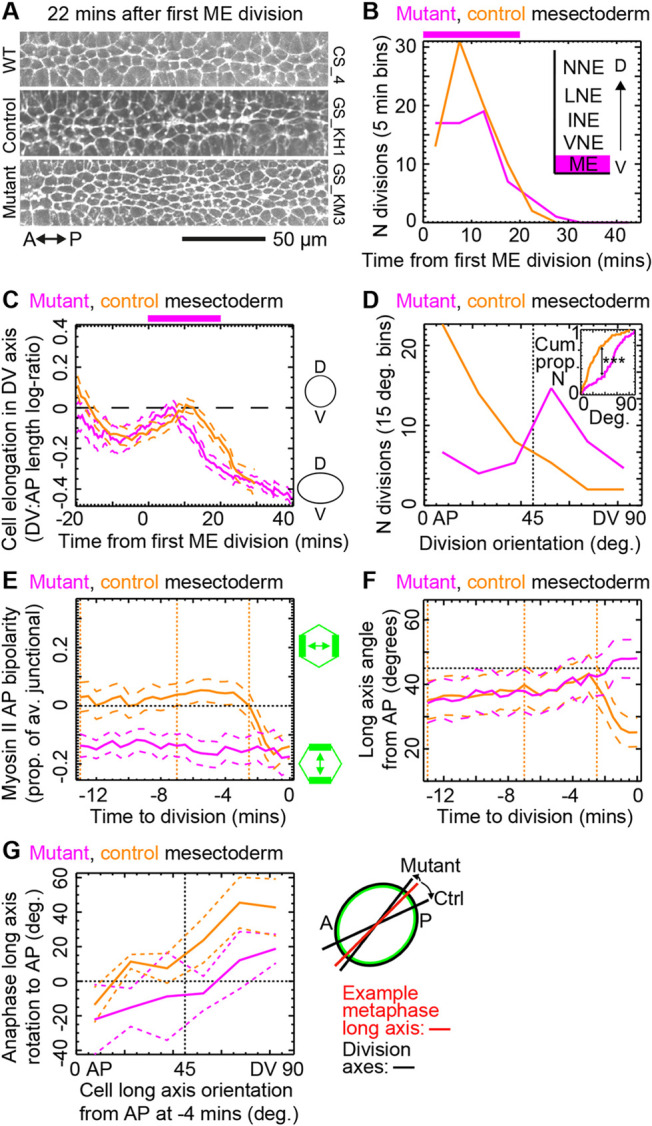
**Anaphase long cell axis rotation towards AP is lost in an AP-patterning mutant.** (A) Stills from E-Cadherin channel for example embryos from WT, *knihb* mutant and control genotypes, showing the width of the mesectoderm (ME) domain after division (see Fig. S5A). (B-G) Data pooled from three *knihb* mutant embryos (magenta) are compared with three control embryos (orange). *N* divisions: mutant, 65; control, 81. (B) Frequency of divisions over developmental time. Top magenta bar shows epoch of ME division. (C) Mutant cell elongation is similar to control over developmental time. Top bar as in B. (D) Frequency of division orientation. Inset shows Kolmogorov-Smirnoff test for orientation difference in cumulative data (***P<0.001). (E) Average Myosin-II planar polarity through mitosis. Controls are indistinguishable from WT (Fig. 4L), whereas mutant ME cells are DV-polarised, probably dominated by strong Myosin-II that develops along the boundary between ME and NE. (F) Angle of cell long axis from AP during mitosis showing the loss of anaphase rotation to AP in the mutant. Compare with WT data in Fig. 4A. Orange vertical lines in E,F show effective start of prophase (−13 min), metaphase (−7 min) and anaphase (−2.5 min). (G) Rotation to AP of cell long axis during anaphase from metaphase (−4 min) angle. Lines should intersect the point [*x*=45, *y*=0] if there is no rotation bias. Control rotates significantly to AP, mutant rotates to DV but not significantly. Control is similar to WT data in Fig. 4H. In C,E-G, dashed lines straddling population means are ±95% CIs.

We observed that ME divisions in the wild type (WT) had the greatest long axis rotation to AP in anaphase (Fig. 4G). We therefore focused on these divisions in the *knihb* and control embryos. We also captured some NNE and early LNE divisions, though in *knihb* these were unlike WT in often being asymmetric in the plane, or out of plane – possibly because apical areas were smaller in the double mutant (Fig. S5B,D). We first checked gross phenotypes, finding that ME divisions in controls were indistinguishable from WT, being predominantly AP-oriented and resulting in an ME domain two to three cells wide after divisions (Fig. 5A; Fig. S5A) and with clear PSBs in the Myosin-II channel. By contrast, *knihb* ME divisions resulted in a four- to five-cell-wide domain, with no visible PSBs, suggesting a randomisation of divisions.

We manually curated automated cell tracking, classified cytokinesis events and aligned cells in space and time in three *knihb* and three control embryos as previously (Fig. S5B-E), identifying 65 double mutant and 81 control ME divisions across the six embryos (Fig. 5B) with high fidelity. The elongation and orientation of ME cells in *knihb* and control embryos was similar (Fig. 5C). However, unlike in the controls, there was no AP-bias to the division orientation in the *knihb* ME (Fig. 5D). Myosin-II planar polarity was DV-oriented (negative) in *knihb* embryos compared with AP-oriented (positive) in the controls during metaphase (Fig. 5E). Importantly, in *knihb* embryos there was no long axis reorientation to AP in anaphase in the ME (Fig. 5F,G). Though not significant, there was a small rotation in the opposite direction (to DV) compatible with the now DV-oriented (negative) Myosin-II planar polarity through mitosis (Fig. 5G). Similar but less marked differences were seen between *knihb* and control cells in NNE and NE cell divisions (Fig. S5F-J).

Overall, we found that when AP-patterning is eliminated, the long axis rotation to AP in anaphase is lost. We conclude that in WT, the long axis rotation to AP in anaphase is due to cues downstream of AP-patterning, correlated with Myosin-II planar polarity, biasing the metaphase spindle towards AP.

### Interaction between mechanics and patterning

We have established that the OCD is a compromise between the cell long axis, which we found can be used as a proxy for the high stress axis, and cues downstream of AP-patterning. We have identified the rotation to AP of the long axis in anaphase (termed ‘anaphase rotation’ below) as a useful measure of this compromise. A large ‘anaphase rotation’ indicates that AP-patterning has successfully pulled the spindle away from the shape long axis in metaphase. Note that this is an apparent rotation only, caused by the spindle taking a different orientation (more often towards AP) to the long axis of the cell at the end of metaphase, resulting in the extension of a new long axis as the spindle separates in anaphase, which is then the orientation of division. We wanted to understand better how a compromise is reached for different combinations of cell elongation and orientation relative to the embryonic AP axis, so we probed the mechanisms of the orienting cues.

**Fig. 6.**
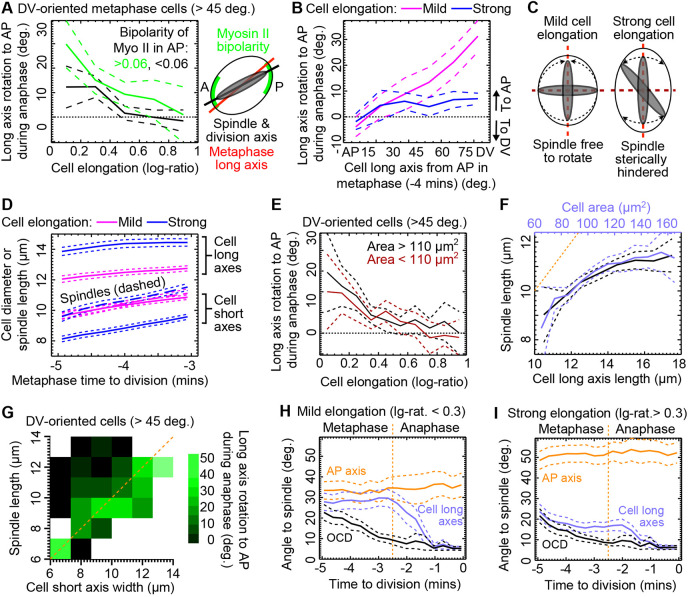
**Interaction between mechanics and patterning.** (A) ‘Anaphase rotation’ for DV-oriented metaphase cells (>45°), separated into high (green) and low (black) myosin AP-oriented planar polarity. All divisions pooled from five Cad/Myo embryos. *N* divisions: Myosin-II <0.06, 160; Myosin-II >0.06, 161. (B) Cell long axes on average rotate to AP during anaphase, attracted by AP-patterning cues. Of cells that are not already AP-oriented, ‘anaphase rotation’ in mildly elongated cells (log-ratio <0.3) is greater than in strongly elongated cells. Cell long axes were measured at −4 min (chosen as a central metaphase time after the spindle has fully formed and before any anaphase-related Myosin-II redistribution to the cytokinesis furrow has begun; Fig. 4L) and at division. *N* divisions pooled from nine WT embryos: mild elongation=359, strong=405. (C) Steric hindrance occurs if the spindle is longer than the short axis of the cell. (D) The evolution of average cell widths and spindle length in metaphase. Data from four Cad/MT embryos are separated into mildly (log-ratio <0.3, *N*=157) and strongly (>0.3, *N*=158) elongated cells at −4 min to division. (E) Rotation of cell long axes to AP in anaphase is strongly dependent on cell elongation, with small rotations in strongly elongated cells. However, this dependence is the same for dividing cells with small (*N*=271) and large (*N*=213) apical areas. Only NE and NNE cells that are DV-oriented (>45° from AP) at −4 min (mid-metaphase) are included from nine WT embryos. (F) Spindle length at −4 min to division increases with the length of the cell long axis (black *x*-axis) and with cell area (blue *x*-axis). For each graph, *N*=315 from all domains of four Cad/MT embryos. Orange line where *y*=*x* for cell long axis length. (G) ‘Anaphase rotation’ on a plot of spindle length versus short axis length, for cells oriented towards DV (>45°) in metaphase (−4 min). Strong rotations occur in cells in which the spindle is less than or equal to the short axis length (orange line, *y*=*x*). See Fig. S6B for total *N*. (H) Angle difference of the cell long axis, AP axis and future OCD from the spindle orientation during mitosis. At anaphase onset (orange vertical line) the cell long axis rotates from its metaphase orientation towards the spindle and future OCD. Data from cells mildly elongated at −4 min to division from four Cad/MT embryos (*N*=157). (I) Same as in H but for strongly elongated cells at −4 min (*N*=158). In all line-graph panels dashed lines straddling population means are ±95% CIs.

First, considering Myosin-II as a proxy for the AP-patterning cues that attract the spindle, we found that cells displaying a stronger AP planar polarisation of Myosin-II had a higher ‘anaphase rotation’ (Fig. 6A). This suggested that it is the local strength, or degree of planar organisation, of AP-patterned cues that attracts the spindle.

Second, we wanted to know whether the cell long axis orientation specifies the choice of cell division axis more strongly in some cells. We have shown that NE cells have less ‘anaphase rotation’ than NNE and ME cells (Fig. 4A,E,G; Fig. S4D), a trend that we noticed was anti-correlated with the strength of cell elongation in metaphase (Fig. 1I). We therefore hypothesised that cell elongation antagonises ‘anaphase rotation’. Supporting this, pooling all divisions from all domains, the metaphase (−4 min) spindle orientation (Fig. S6A) and the future OCD (Fig. 6B) were better aligned with the cell long axis in metaphase (−4 min) if the cell was strongly elongated (log-ratio>0.3). This suggests that cell elongation constrains the spindle orientation away from short cell axes (Fig. 6C).

Comparing the lengths of cell long and short axes with the metaphase mitotic spindle lengths (Fig. 6D), we found that the average length of the spindle was similar to the short axis width in mildly elongated cells, giving the spindle room to rotate. However, for strongly elongated cells, their short axis widths were on average significantly shorter than the spindle length, suggesting that mitotic spindles in these cells were sterically hindered away from short axes. Pairing the mitotic spindle length of each cell to its short axis lengths, we found that most mitotic spindles were indeed longer than the short axis of the cell (Fig. S6B). Thus, in most cells, the spindle was longer than the cell short axis, preventing the spindle from responding freely to AP-patterning cues.

To understand further the nature of this hindrance, we predicted that when comparing cells of the same cell elongation ratio but with different apical areas, larger cells would have more space for the spindle, which should be able to rotate further from long axes. Surprisingly, separating divisions into approximately equal populations with small or large apical areas (threshold 110 µm^2^) in metaphase (−4 min), we found no difference in the amount of ‘anaphase rotation’ in cells of the same elongation ratio (Fig. 6E), suggesting that somehow spindle behaviour was scaling with cell size. Indeed, spindle length was not the same between cells in metaphase, increasing instead with cell long axis length and hence also cell area (Fig. 6F). Confirming that mitotic spindle length tracked raw long axis length rather than cell shape anisotropy, the spindle length was not correlated with cell elongation ratio (Fig. S6C). Spindle length appeared to plateau at ∼11 µm in larger cells, suggesting that this is the unconfined spindle length, below which spindles are adapting to confinement. Thus, in all cells except those with the largest apices, the spindle adapts to the size of the cell long axis. Any cell shape anisotropy would mean that the cell short axis will be shorter than the spindle, sterically hindering its movement (Fig. 6G; Fig. S6B,D).

Finally, as mitotic spindles were dynamic within the plane of the epithelium during metaphase (Movie 7) (Scarpa et al., 2018), we asked whether these movements lead to a progressive improvement in the fit of the spindle with mechanical and patterning cues. Interestingly, for mildly elongated cells, we found no evidence that the mitotic spindle on average improved its orientation relative to AP-patterning and cell elongation cues over the course of metaphase, despite some change in spindle orientation as it settled on the division orientation (Fig. 6H). This was compatible with no change in the average planar polarity of Myosin-II (Fig. 4L) and with comparable increases in both spindle and cell long axis lengths (Fig. 6D) through metaphase. For elongated cells, however, the spindle initially improved its alignment with the cell long axis at the expense of alignment with AP (Fig. 6I), suggesting that AP-patterning is important for the initial orientation of the spindle but, as it settles in the plane, steric hindrance exerted by the cell short axis ensures that it adjusts towards the long axis in elongated cells.

## DISCUSSION

### Mechanics re-orients OCD during metaphase

**Fig. 7.**
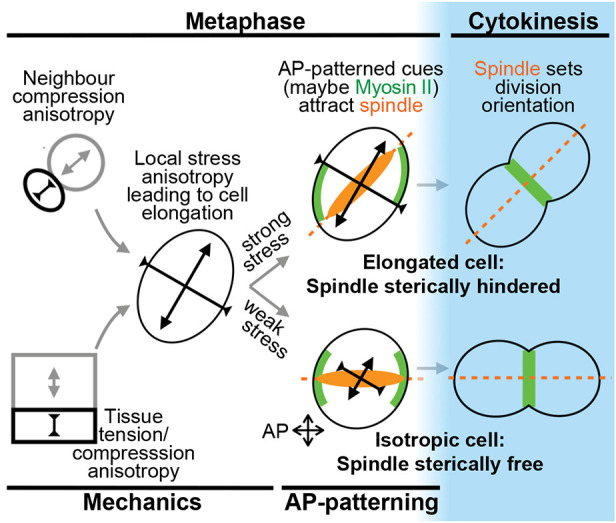
**Orientation of planar division.** Summary of how mechanical tension and compression, from neighbouring tissues and dividing cells, combine with AP-patterning in metaphase to orient the mitotic spindle, and hence the orientation of planar division in a *Drosophila* embryonic epithelium.

Our work shows that, in the *Drosophila* embryo, epithelial cells can undergo a re-orientation of the axis of highest stress during metaphase, which results in a re-orientation of their OCD. Metaphase stress re-orientation can occur due to temporary compression both from a neighbouring tissue or from neighbouring divisions (Fig. 7).

The metaphase spindle length is frequently longer than the metaphase short axis length, sterically constraining the spindle away from the short axis. The length of the mitotic spindle is variable, correlated with the length of the cell long axis, suggesting that the spindle adapts its length to the longest cell axis, as has been shown in other tissues (Hubatsch et al., 2019; Rieckhoff et al., 2020). Except for the largest cells, in which the full-length spindle (∼11 µm) appears to rotate freely, any cell shape anisotropy will result in the spindle being longer than the cell short axis, resulting in steric hindrance. In the germband, therefore, stress orients the OCD indirectly through metaphase cell shape, though we cannot rule out that direct stress sensing is also in play (Lisica et al., 2022; Minc et al., 2011).

The control of planar OCD differs between tissues (Ramkumar and Baum, 2016; Tarannum et al., 2022; Taubenberger et al., 2020). During cycle 14 divisions the *Drosophila* embryonic epithelium is an immature epithelium with adherens but not septate junctions and lacking cell–extracellular matrix (ECM) adhesions (Tepass et al., 2001). Active forces during cell division are therefore readily expressed as changes in cell shape. In this tissue, NE cells are columnar, increasing their apical width by 50% before anaphase to accommodate planar rotation of the mitotic spindle. Even after mitotic rounding at the end of metaphase, the average cell elongation remains strong (1.4:1 aspect ratio). By contrast, divisions in epithelia with less columnar cell shapes (Woolner and Papalopulu, 2012), with stronger adhesions (Lisica et al., 2022) or ECM (Bosveld et al., 2016), show less dramatic deformations throughout mitosis. These, and tissues with small mitotic spindles relative to cell width (Woolner and Papalopulu, 2012), may need additional mechanisms to detect stress anisotropies.

### Temporary tissue compression

By measuring tissue-scale tension ratios using laser ablation, we and others (Collinet et al., 2015) have found that DV tissue stress exceeds AP stress for most of the developmental period we studied, resulting in DV-elongated cells. However, we identified a ∼20-min period when LNE cells become temporarily elongated in AP (Fig. 2H,I). The first LNE divisions soon after are AP-oriented on average (Fig. 2G), compatible with their OCD being determined by a combination of metaphase cell shape and AP-patterning. Our box laser ablations reveal a strong correlation between cell elongation and the axis of high cell stress. Therefore, considering cell elongation as a proxy for local stress anisotropy, the observed change in cell orientation provides evidence of a temporary change in the orientation of tissue stress.

What could be causing the temporary AP-orientation of LNE cells? We observed a synchronous decrease in LNE cell area and increase in cell area in the dorsally adjacent NNE, as the LNE cells re-orient to AP (Fig. 2I), suggesting that the switch in cell orientation from DV to AP could be caused by a compression from the NNE domain in which cells are dividing. Alternatively, a temporary release of tissue-extrinsic DV tension could be explained by the increase in compliance of the dorsal amnioserosa tissue (Pope and Harris, 2008). However, this would not explain why the loss of DV stress is temporary. We therefore favour the hypothesis that mitotic divisions in the NNE domain flanking the LNE in DV is directly responsible for the AP-orientation of LNE cells, compressing apices in DV, but more work is needed to confirm the nature of this stress change.

### Control of OCD by AP-patterning

We show that cues downstream of AP-patterning bias the metaphase spindle towards AP across the whole tissue (Fig. 7). This generalises our previous finding that strongly Myosin-II-enriched PSBs restrict the mobility of the closest mitotic spindle pole (Scarpa et al., 2018). We have measured Myosin-II planar polarity as a proxy for AP-patterning and show that in an AP-patterning mutant, both the AP-oriented Myosin-II planar polarity and the mitotic spindle bias to AP are lost. Though it is possible that Myosin-II itself could be involved in re-orienting the mitotic spindle, possibly through increased cortex stiffness (Spiro et al., 2014), we do not address this directly.

The AP-patterning system straddles the whole DV circumference of the embryo (Irvine and Wieschaus, 1994), providing a mechanism for how even the ventral ME domain has AP-polarised Myosin-II before and during divisions (Fig. 4J,L). Previous reports suggest that an extrinsic AP-oriented stress drives AP-oriented divisions in the isotropic ME cells (Wang et al., 2017) and that this operates through Pins polarisation along AP (Camuglia et al., 2022). However, the loss of ‘anaphase rotation’ and randomisation of OCD in our AP-patterning mutant ME cells is compatible with AP-patterning directing the OCD in these cells. If so, this would require the emergence of Pins planar cell polarity in metaphase to be downstream not only of stress anisotropy, as clearly demonstrated for divisions in the embryonic head (Camuglia et al., 2022), but also of AP-patterning cues. Precisely how AP-patterning, stress anisotropy and steric hindrance of the mitotic spindle combine to interact with Pins and whether this varies between epithelial domains (Scarpa et al., 2018) requires further work.

Our results show that AP-patterning biases oriented cell divisions to AP, which could contribute to extending the AP body axis in cooperation with cell rearrangements, if not over-ridden by mechanics. In vertebrate models, both cell rearrangements and cell divisions contribute to extending the AP axis during axis extension, with the loss of PCP randomising both rearrangements and divisions in *Xenopus*, zebrafish and mouse models (Morin and Bellaiche, 2011). The actin-nucleator Diaphanous is implicated in the mechanism for how PCP ensures AP-oriented divisions (Castanon et al., 2020), but how mechanics interacts with PCP in these vertebrate examples is an interesting open question.

### Interaction between mechanics and patterning

We found that discrepancies between the orientations of local stress and AP-patterning are resolved in metaphase by the positioning of the mitotic spindle, which in turn sets the perpendicular location of the cytokinesis ring and hence the OCD (Dimitracopoulos et al., 2020). We observe that strong local stress anisotropy can override AP-patterning by imposing cell shape changes that limit the movement of the mitotic spindle during metaphase. Conversely, the extent of apparent rotation of the cell shape long axis at anaphase reveals how much the stress axis has been overridden by AP patterning. Indeed, we find that mildly elongated cells, or cells with large apices, readily orient their divisions to AP. In line with these findings, we have previously shown that mildly elongated cells abutting the parasegments, which are enriched in Myosin-II, orient their divisions towards AP, whereas highly elongated cells, even when abutting a parasegment, do not (Scarpa et al., 2018). In addition, in the posterior-most germband domain, cell elongation is on average AP-oriented in the same orientation as AP-patterning, doubly ensuring AP-oriented divisions (da Silva and Vincent, 2007). Overall, AP-patterning on its own would result in AP-oriented divisions, contributing to axis extension, but greater DV tension along most of the body axis overrides patterning, leading to predominantly DV-oriented divisions.

In summary, we find that the Hertwig rule, which states that the interphase cell elongation sets the OCD (Hertwig, 1884), can be violated in four ways in the *Drosophila* embryonic epithelium: (1) if the cell long axis is re-oriented in metaphase by compression from neighbouring tissues, this can re-orient the OCD; (2) compression from neighbouring cell divisions during metaphase can also re-orient the OCD; (3) the mitotic spindle in all cells is attracted towards the embryonic AP axis by AP-patterning cues, orienting the spindle away from cell long axes; (4) for cells where the mitotic spindle does align well with cell shape, this is caused by steric restriction of spindle movements by the cell short axis in metaphase. Our data suggest that the mechanical and patterning control of OCD in this tissue do not require a memory of interphase shape, but rather is set by a combination of instantaneous mechanical stress and AP patterning during metaphase (Fig. 7).

## MATERIALS AND METHODS

### Embryo imaging

#### Fly stocks

For wild-type live imaging, automatic tracking of cell divisions and fluorescence quantification, we used the stocks *sqh*^*AX3*^*; (endo)DE-Cadherin-GFP, sqh-mCherry* (abbreviated as ‘Cad/Myo’) and (*ubi)DE-Cadherin-GFP; jupiter-mCherry* (abbreviated as ‘Cad/MT’). *(endo) DE-Cadherin-GFP* (Huang et al., 2009) is a knocked-in tagged version of the endogenous *shotgun* gene encoding DE-Cadherin, whereas (*ubi) DE-Cadherin-GFP* is a transgene version of the same gene expressed under the control of the ubiquitin promoter (Oda and Tsukita, 2001). Both are located on the second chromosome. *sqh-mCherry* (Martin et al., 2009), also located on the second chromosome, is a transgene version of the gene *spaghetti-squash* (*sqh),* encoding the Myosin-II Regulatory Light Chain (MRLC). We use it here in a *sqh*^*AX3*^ null mutant background so that all the MRLC molecules are labelled with mCherry. We made the *(endo)DE-Cadherin-GFP, sqh-mCherry* recombinant chromosome using standard *Drosophila* genetics. *Jupiter-mCherry* labels the MT (Bergstralh et al. 2015).

To remove antero-posterior patterning in the embryo trunk, we used the double mutant *kni*^*10*^*, hb*^*4*^ (null mutants for the gap genes *knirps* and *hunchback*), for which we have evidence that polarised cell intercalation is abolished throughout the trunk [figure S3G in Butler et al. (2009)]. To visualise Myosin-II and cell contours in this double mutant, we used a *sqh-EGFP*^*29B*^*, Gap43-mCherry* recombinant on the X chromosome (Lye et al., 2024). *Sqh-EGFP*^*29B*^ (Ambrosini et al., 2019) is a knocked-in tagged version of the endogenous *sqh* gene, so all MRLC molecules are labelled with GFP. *GAP43-mCherry* (Izquierdo et al., 2018) labels the cell contours*.* We made the stock *sqh-EGFP*^*29B*^*, GAP43-mCherry; ; kni*^*10*^*, hb*^*4*^*/TM6B* and used the homozygous mutant embryos *kni-, hb-* in the progeny for the experiments (abbreviated as ‘*knihb*’). Mutant embryos were identified by lack of cell intercalation and AP-stretched cell shapes. The remaining sibling embryos were used as controls.

For tissue-scale laser ablation experiments, we used the stock *sqh*^*AX3*^*; sqh-GFP42; GAP43-mCherry/TM6B* (Tetley et al., 2016) and for the ‘cut-out’ laser ablation experiments, we used the recombinant stock *sqh-EGFP*^*29B*^*, GAP43-mCherry* mentioned above.

#### Live imaging

Dechorionated embryos were transferred into halocarbon oil (Voltalef PCTFE, Arkema), mounted on a stretched oxygen-permeable membrane with their ventral side facing up and covered by a coverslip which was supported by a single coverslip bridge on either side of the membrane. Imaging was performed using a Nikon Eclipse E1000 equipped with a spinning disk unit (Yokogawa CSU10), laser module with 491 nm and 561 nm excitation (Spectral Applied Research LMM2), and a C9100-13 EM-CCD camera (Hamamatsu). Image acquisition was carried out using the Volocity software (PerkinElmer). The imaging temperature was 21±1°C.

For live imaging, movie volumes ∼30 µm deep were taken for 50-100 min covering cycle 14 divisions, always starting before the first ME division. Image volumes were sampled every 1 µm in depth and acquired every 30 s for Cad/Myo, *knihb* and control movies. Cad/MT image volumes were sampled every 0.7 µm in depth and every 20 s in time to image mitotic spindles and centrosomes. The *xy* pixel size throughout was 0.364 µm, with the AP field of view 254 µm from immediately posterior of the cephalic furrow (never in view, Fig. 1A), covering approximately three thoracic and the first two to three abdominal parasegments. Five WT Cad/Myo movies CS_1 to CS_5 correspond to ‘080116’, ‘130116’, ‘160518’, ‘170518’ and ‘251116’, respectively, as used in Scarpa et al. (2018).

### Tracking cell shapes in 2D and 3D

#### Cell tracking at and below the level of adherens junctions

Cell apices in movies were segmented and linked in time in an iterative process using an adaptive watershed algorithm (Blanchard et al., 2009; Butler et al., 2009) written in IDL (NV5 Geospatial). Briefly, the surface of the embryo was quantified as a smoothed ‘blanket’ spread over the apical-most junctional fluorescence in E-Cadherin movie channels. Cell segmentation and tracking was performed on curved planes at a constant depth relative to this embryo surface, with median and rolling ball filters applied as necessary to optimise subsequent cell tracking. For tracking at the level of AJs, a projection of local depths centred on 2-3 µm below the embryo surface was used (Fig. 1B; Fig. S1A). Manual corrections were further performed on movies CS_2, CS_3 and CS_4 and all *knihb* and control movies. Manual supervision focused particularly on dividing cells and their immediate neighbours. In Cad/MT movies, the higher expression levels of E-Cadherin resulted in higher fidelity automated cell segmentation and tracking, reducing the benefit of manual curation.

Cell segmentation and tracking was imperfect if Cadherin fluorescence was locally weak, if the smooth epithelial surface ‘blanket’ was unable to follow cell apices into furrows, such as at the VML before it was fully closed, and at the edges of images. We therefore removed partial cells at the movie edges and those with extreme or unlikely cell centroid jumps or cell shape changes (e.g. as seen in the first few frames of Movie 1) from all subsequent analyses.

The number of cells successfully tracked over time per embryo is shown in Fig. S1D,E. For each cell at each time point, coordinates of cell centroids, perimeter shapes, bi-cellular (BCJ) and tri-cellular (TCJ) junction indices, and links forwards and backwards in time were stored.

#### 2D cell shape analyses

Cell shapes were approximated by best-fit ellipses. Cell apices were first un-tilted and un-curved according to the surface normal and principal curvatures of the local tissue surface, respectively. Best-fit ellipses were then found by minimising the area of mismatch between pixelated contours and ellipses, with constraints that the fitted ellipses have the same area and centroid as the cell contours.

The log-ratio of DV to AP cell lengths (Fig. 2F,I) or cell lengths along the future OCD (Fig. S3E) were calculated by projecting cell shape ellipses onto the relevant axis orientations, giving diameters of the ellipses in those orientations.

### Identifying cell division events

#### Automated identification of cell divisions

We defined the completion of cytokinesis as the timepoint at which the mother cell splits into two daughter cells, marked by the appearance of a new segmented E-Cadherin interface between daughter cells. However, this alone is not a robust enough rule to identify cytokinesis events reliably because oversegmented cells can appear as two cells divided by a false interface. Through trial and error, we evolved a set of algorithmic rules to identify cytokinesis events robustly in segmented and tracked cell data (Fig. 1).

Each rule was computed for each cell in each movie frame and assigned a value between 0 and 1. These rules set the minimal change (gradient) in shape and fluorescence expected in the mother cell in the 10 min before the completion of cytokinesis (see Fig. 1F). Gradients above the minimal gradient all evaluated to 1. For some rules this minimal gradient was effectively a threshold, with the rule evaluated to 1 above and 0 below. For other rules that were more likely to be broken (for example, the dumbbell shape just before cytokinesis is not guaranteed), though in rare circumstances, the rule gradually evaluated to 0 with decreasing gradient according to a ‘half-range’ parameter, the gradient value at which the rule evaluated to 0.5 (Table 1).

**Table 1.**
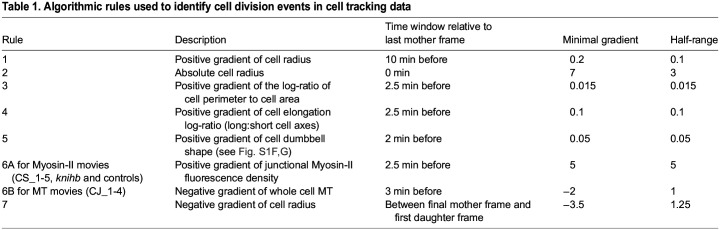
Algorithmic rules used to identify cell division events in cell tracking data

A composite cell division probability is then calculated by multiplying all rule values together. This method ensures that most but not all rules must be met, allowing for some unexpected division behaviour. Looking at distributions of the above rule values for example divisions, along with visually minimising false positives and false negatives, we set a threshold composite probability of 0.01, above which cells were identified as dividing. We reasoned that missing data from the occasional missed division was less bad than including false data from the occasional false division.

To assess the success of this method we visually confirmed that there were no false positives (see all cell divisions identified for embryo CS_4 in Movie 2) and compared cell divisions identified with the above algorithmic classifier with manually identified cell divisions.

#### Manual identification of cell divisions

All visible cell divisions were manually located at the frame closest to cytokinesis completion in two movies in which cells had been tracked with automated methods only (CS_1 and CS_5) and two movies in which cell tracking had also been manually curated (CS_2 and CS_4). *xy* frame coordinates of manually identified cytokinesis events were compared with cell divisions identified with automated methods and the results shown in Table 2. Around 50% of divisions were correctly identified in the first two movies, rising to over 90% for manually curated movies. Failure to identify cell divisions was almost always due to interruptions in the tracking of mother cells in the previous 10 min, or due to mistracked daughter cells immediately after division. In only a handful of cases did the above algorithmic rules fail to identify division when cells were correctly tracked. In the four movies we checked, with a total of 455 divisions, only two false positives were found.

**Table 2.**
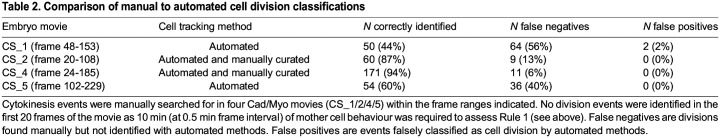
Comparison of manual to automated cell division classifications

The E-Cadherin channel in Cad/Myo movies is endogenously tagged E-Cadherin-GFP (Huang et al., 2009), whereas in Cad/MT movies E-Cadherin-GFP was driven by the ubiquitin promoter (Oda and Tsukita, 2001). As a result, the E-Cadherin signal was stronger and automated cell segmentation and tracking was more reliable in the Cad/MT movies, so manual curation was less necessary.

For *knihb* and control movies, cell shapes were tracked using the membranous GAP43-mCherry signal and all were manually curated. In the six *knihb* and control movies, a total of 311 divisions were correctly identified. Note that the number of cell divisions classified in each movie depends on the particular field of view, when the movie starts and finishes (Table 3, cells must be tracked for 10 min before cytokinesis and the two daughters must be recognised within a few frames after cytokinesis in order to be classified) and whether the embryo rolls in the field of view. For all embryos from all genotypes we generated movies of all individual cell divisions (as in Movie 2 for CS_4) and manually checked for false positives and removed these cells from all analyses (Table 3).

**Table 3.**
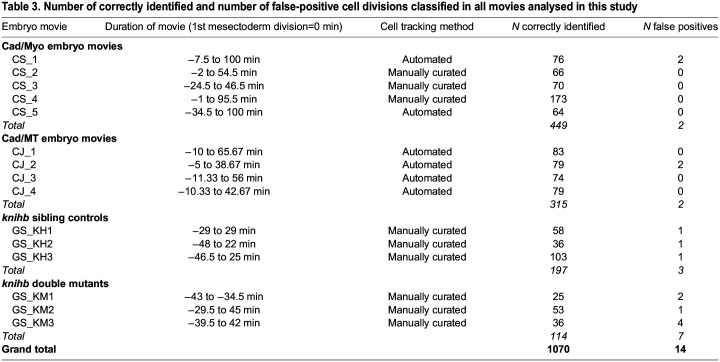
Number of correctly identified and number of false-positive cell divisions classified in all movies analysed in this study

#### Cell division tracking summary

With the above methods we identified 450 divisions in five Cad/Myo movies, 286 divisions in four Cad/MT movies, 114 divisions in three *knihb* movies and 197 divisions in three control embryos. All cell divisions were cycle 14 divisions captured between 0 min and 100 min after the first ME division, straddling stages 8 to 10 (Bate and Martinez Arias, 1993).

Each dividing cell was then assigned its own ‘time to division’ axis, with the frame after which the mother splits into two daughters set to 0 min.

### Embryonic time and space axes

Embryo movies were co-aligned according to a set of temporal and spatial developmental landmarks so that data from different movies and different genotypes could be overlaid and compared directly.

#### Movie staging and synchronisation

The temporal developmental origin (0 min) in all movies of all genotypes was set to the completion of the first observed ME cytokinesis. This is equivalent to ∼35 min after the start of axis extension (Butler et al., 2009; Tetley et al., 2016) and marks approximately the transition from the fast to slow phase of axis extension (Irvine and Wieschaus, 1994) as the cell intercalation strain rate slows down (Butler et al., 2009).

We used all data between −20 min (mid developmental stage 7; Bate and Martinez Arias, 1993) and 100 min (mid stage 10) in our WT movies, each of which covered a subset of this range (Fig. S1D,E). Within this range, ∼0-30 min represents stage 8 and 30-75 min is stage 9. Note that our imaging temperature of 21°C is lower than most early staging reports that were imaged at 25°C, hence development is slower. Because AP-patterning gene expression changes from primary pair-rule (stage 7) to secondary pair-rule (stage 8) to segment polarity genes (stages 9-10) over this time range (Clark and Akam, 2016), we were careful throughout all analyses to be clear from which developmental stage data is presented.

#### DV coordinates and domains

DV cell location was set in µm from the VML at 15 min after the first ME division, which is when DV tissue convergence stalls across movies (Fig. S2A). Cells carried these DV coordinates forwards and backwards in time, defining a co-moving coordinate system. This coordinate system was in turn used to classify five separate cell-type domains in DV in each movie so that we could pool cells from different movies into the same cell types for analysis.

The most ventral cells were easily distinguished as ME cells by their early cell divisions (complete by 20 min) and small AP-oriented shapes thereafter. The ME occupied a region ∼0-12 µm from the VML. On the dorsal side of the NE, the NNE cells were identified from their early cell divisions, which were also complete by 20 min. A line at 66 µm from the VML separated the NNE from the NE quite accurately in all movies (pooled in Fig. S2B,C).

The NE was broken down into three domains, VNE, INE and LNE, that from cell counts of *in situ* images of *ind* expression (Lye et al., 2024) map approximately to *vnd*, *ind* and *msh* expression domains (Cornell and Von Ohlen, 2000; Reeves and Stathopoulos, 2009). *vnd* cells are unique in becoming progressively elongated in DV and larger, occupying a band between the border with the ME at 12 µm and 30 µm. We distinguished *ind* from *msh* domains from their different cell division timings [mitotic domains 21 and *N* in Foe (1989), respectively]. This allowed us to identify the approximate dividing line at 48 µm from the VML, which consistently separated LNE from INE divisions in all movies (pooled in Fig. S2B,C and Fig. 2B).

To measure angles relative to embryonic axes, the orientation of the VML over time for each movie was used as the evolving orientation of the AP axis. Note that some embryos rolled a little in the DV axis in the field of view, but this is corrected for by keeping track of the VML as it rolled.

#### Landmarks of mitosis

In Fig. 1K we relate our measured average changes in morphology and fluorescence during mitosis to known mitotic landmarks and checkpoints as reviewed by Ramkumar and Baum (2016). We do not know when the G2/M (interphase/prophase) transition occurs in our data, corresponding to the rise in CDK1-cyclin B activity, but our first measurable change associated with mitosis is a drop in apico-medial Myosin-II at −13 min. We therefore call this the effective onset of prophase in our data.

Prometaphase is defined as starting with NEBD and ends with spindle formation as metaphase commences. We see two Jupiter-mCherry centrosomal foci below each nucleus during prophase, which disappear at around −7.5 min, which we define as NEBD. In a separate study, NEBD was also seen to occur at ∼−7 min in the germband from the loss of mTor-YFP, a nuclear envelope marker (Lye et al., 2014). A variably oriented elongated spindle then appears almost immediately, so we set the onset of metaphase in our data to −7 min.

The spindle assembly checkpoint (SAC) is defined as just before the onset of spindle separation, with associated cell elongation, and marks the transition to anaphase. We see that anaphase cell elongation starts after −3 min and the cytokinesis ring appears at −2.5 min in our data (Fig. S1N,O), so we set the SAC to −3 min.

### Laser ablation

#### Tissue scale and cut-out box ablations

Laser ablation experiments were performed using a TriM Scope II Upright 2-photon Scanning Fluorescence Microscope controlled by Imspector Pro software (LaVision BioTec) equipped with a tuneable near-infrared (NIR) laser source delivering 120 femtosecond pulses with a repetition rate of 80 MHz (Insight DeepSee, Spectra-Physics). The laser was set to 927 nm, with power ranging between 1.05-1.70 W. The maximum laser power reaching the sample was set to 220 mW and an Electro-Optical Modulator (EOM) was used to allow microsecond switching between imaging and treatment laser powers. Laser light was focused by a 25×, 1.05 Numerical Aperture (NA) water immersion objective lens with a 2 mm working distance (XLPLN25XWMP2, Olympus). Ablations were carried out during image acquisition (with a dwell time of 9.27 µs per pixel), with the laser power switching between treatment and imaging powers as the laser scanned across the sample. For tissue-scale cuts, targeted line ablations of 20 µm length were performed in an AP or DV orientation using a treatment power of 220 mW on the *vnd* domain (rows of cells 2, 3, 4 from the VML) of the ventral neuroepithelium of stage 9 embryos. Embryos were staged by waiting for midline closure after the last division in the ME (mitotic domain 14), equivalent to 25-30 min after the first ME cell division. Images were acquired with a frame delay of 1 s for tissue-scale cuts and 753 ms for cut-out ablations.

#### Analysis of ablation recoil velocity

To quantify recoil velocity, we automatically detected the position of the ablation line in the images, chose a region of interest around it, detected the fluorescent structures inside this region and inferred their motion from the video sequences. The region of interest in each movie is defined as a 30 pixel area on each side of the cut. The position of the ablation line was recorded by taking a screen snapshot at pre-acquisition. The screenshot was cropped and the site of cut automatically matched to its location in the video sequence. To analyse recoil velocities, we first performed two pre-processing steps: a frequency filter to remove stripes caused by electrical interference in the detector and a median filter denoising step. Subsequently, we computed the optical flow in order to estimate the velocity of the structures using the algorithm described in Brox et al. (2004) in the whole region of interest and on the bright pixels of signal in the region of interest for each embryo. To use the flow of Myosin-II to measure recoil away from the cut, only the velocity component of flow perpendicular to the cut is considered in the analysis. For each side of the cut, the average normalised relaxation speed is computed and the two averages are combined to give a final normalised relaxation speed away from the cut (Fig. 2E). This final velocity was compared between the two conditions with an unpaired two-tailed Mann-Whitney test.

#### Analysis of ablation cell shapes

For cut-out laser ablations, 43×43 µm (255×255 pixels) movies were focused around a central cell. Cell shapes were analysed at five timepoints (−3.77 s) before ablation and 20 timepoints (15.1 s) after ablation. The outline of each cell was manually traced over the Sqh-eGFP channel using the polygon tool in Fiji and its best fit ellipse was calculated. Shape descriptors (long axis, short axis, log-ratio of long:short axis) were calculated from the best fit ellipse, and the area of cells were measured from manually traced polygons. The AP axis of each embryo was located by taking a low magnification image.

### Fluorescence intensity

#### Fluorescence normalisation

For each cell in each movie frame we quantified the density of fluorescence in each movie channel associated with membrane (junctional) and cytoplasmic (medial) domains. Bleaching of Sqh-mCherry, endo-E-Cadherin-GFP, ubi-E-Cadherin-GFP and to a lesser extent Jupiter-mCherry, was significant over the 50-100 min of each movie, so we normalised the greyscale range of each channel in each movie frame. For example, for endo-E-Cadherin-GFP, we set the lowest 20% of pixels as background (0 greyscale) and pinned the 99.5th percentile to 200 greyscale, allowing the top 0.5% to occupy the top 55 greyscale with minimal overexposure at 255. Background and signal percentiles for all movies are shown in Table 4.

**Table 4.**
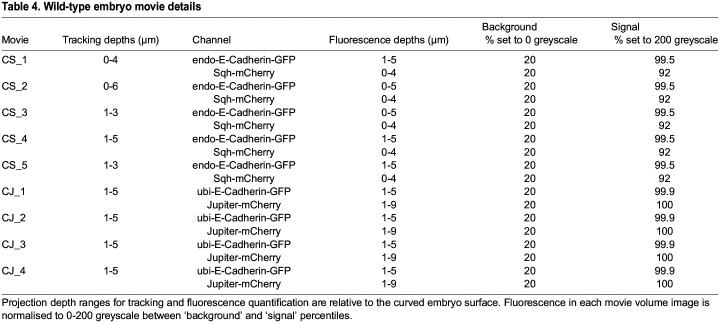
Wild-type embryo movie details

#### Junctional and medial fluorescence quantification

Junctional and medial domains were determined by the outline coordinates of each cell, as recorded by cell shape tracking. The coordinates of non-overlapping pixelated cell perimeters were stored by the cell tracking for each cell in each movie frame. The fluorescence intensity of a particular movie channel at cell perimeters was quantified as the average of cell perimeter pixels and neighbouring pixels to a width *w*=0.65 µm perpendicular to the local perimeter, meaning the perimeter pixel and one pixel either side. For the Myosin-II channel, this was a compromise between being wide enough to encompass all junctional Myosin-II fluorescence but narrow enough to minimise the inclusion of medial Myosin-II. We then summarised the fluorescence intensity of every cell–cell junction as the average intensity of all perimeter pixels along the junction.

Fluorescence density at TCJs is expected to be 1.5× that of BCJs because of the convolution of the confocal point spread function of three junctions meeting. This extra fluorescence at TCJs contributed by the neighbouring cell BCJ pollutes attempts to summarise the polarity of membrane fluorescence, particularly in elongated cells in which vertices tend to be clustered towards the cell long ends (Bosveld et al., 2016; Scarpa et al., 2018). We defined TCJs as pixels within 0.65 µm radius of the TCJ centre point, allowing us to remove these and define a ‘no-vertex’ BCJ density (see ‘Top-down view’ in Fig. S4E). For Myosin-II channels, the fluorescence density of each cell–cell interface in each movie frame was calculated as the average intensity of ‘no-vertex’ BCJ pixels.

#### Portraits of average cell fluorescence through mitosis

We set out to build up a portrait of the behaviour (shape and fluorescence) of the average cell in 3D through mitosis. To do this, we needed to overlay and orient cells in the same way so that we could average the fluorescence surrounding cells in both movie channels in Cad/Myo and Cad/MT movies.

We first superimposed all cells by translating the centroid of each cell (from AJ level tracking) to the origin, bringing along both the local embryo surface normal and a local 3D volume of fluorescence from both movie channels. Secondly, we rotated the local fluorescence volumes so that the local surface normal of each cell aligned with the positive *z*-axis, meaning that cell bases extended from the cell centroid in negative *z*. Finally, we rotated the volumes around the *z*-axis to set the future OCD to the *x*-axis. With all cells overlaid, standing upright and dividing along *x*, we were able to take the average voxel fluorescence intensity in the surrounding image volumes: 411 ectoderm cell divisions were overlaid from Cad/Myo embryos and 270 from Cad/MT embryos.

Cutting this averaged fluorescence volume at *z*=0 gives the shape and fluorescence of the averaged cell at the AJ level (top row of Fig. S1N,O; second panel of Movies 3 and 4). Cutting at *z*=−4 shows the average cell behaviour at a sub-AJ level (bottom row of Fig. S1N,O; fourth panel of Movies 3 and 4). Despite individual mitotic cell behaviour being variable (Fig. S1M; Movies 2 and 7), this method yields smooth average mitotic behaviour, separated into clear medial and junctional fluorescence, with horizontal elongation in the future OCD and a vertical cytokinesis ring. The similarity in endo-E-Cadherin and ubi-E-Cadherin fluorescence patterns between the Cad/Myo and Cad/MT average cells allows comparison of the Myosin-II with the MT patterns at different depths.

#### Myosin planar polarity

To quantify the apical junctional planar polarity of Myosin-II, we used the ‘no-vertex’ BCJ measures from above. We first expressed BCJ fluorescence intensity around each cell perimeter as a function of planar angle around the cell centroid. Treating this intensity signal from 0-360° as a periodic repeating signal, we calculated its Fourier decomposition, extracting the amplitude of the period 2 component as the strength of Myosin-II bipolarity or planar polarity, with its phase representing the orientation of cell planar polarity. To control for variation in Myosin-II intensity between embryos and across the field of view, and due to possible bleaching over movie time, we normalised the strength of planar polarity by dividing the Fourier amplitude by the mean cell perimeter Myosin-II signal, so that planar polarities were comparable across images, frames and embryos.

Tensors describing the orientation and strengths of the principal axes of Myosin-II planar polarity were projected onto embryonic axes in a similar manner to the ‘2D cell shape analyses’ section above.

Using the above methods, we produced planar polarity measures projected along the AP axis for each cell at each time point, that were independent of each other and normalised to control for variation in Myosin-II fluorescence.

#### Jupiter fluorescence quantification

We quantified average Jupiter fluorescence at junctions and medially across depths 1-9 µm from the tissue surface (Fig. S1K,L).

#### 3D spindle tracking

To quantify spindle depth and rotation accurately, we applied an adaptive binary fluorescence threshold to distinguish Jupiter pixels from Jupiter-free cytoplasmic pixels (Fig. S1L, Fig. S4A). Principal component analysis (PCA) of the 3D clouds of thresholded Jupiter pixels provided ellipsoid fits to spindle shapes for each cell (Fig. 4C). The shape of the spindle identified in this way was essentially linear, dominated by its principal component, with much smaller widths orthogonal to the spindle long axis (Fig. S4B).

### Constructing an anisotropic neighbour compression stress tensor

Cell rounding in prophase and metaphase, and cell elongation during anaphase, are actively driven by increased cortical actomyosin activity in dividing cells (Ramkumar and Baum, 2016; Rosa et al., 2015). The rounding and elongation would therefore be expected to exert outward forces on neighbouring cells, compressing them. Cells under isotropic planar compression can be forced to divide out of the plane, but we were more interested in anisotropic compression that would influence planar cell elongation in metaphase, constraining the spindle orientation and hence the OCD (Fig. 3D-J; Fig. S3E-G).

We therefore set out to construct a summary measure of the anisotropic compression that cells are under from surrounding mitotic cells, making the simplifying assumption that the enlargement of cells during mitosis is a proxy for the strength of compression they exert on neighbours. That is, we assumed that the active mitotic rounding force increased with how rounded cells became through prophase to metaphase. During anaphase, elongation along the future division axis also exerts a pressure in the direction of elongation (Gupta et al., 2021). We used the approximately linear increase in radius of cells in prophase to metaphase and the elongation of cells in the OCD during anaphase (Fig. S3E) to estimate the likely compressive force exerted (Fig. 3E).

Then for each cell in interphase and mitosis we constructed a compression tensor by adding the compressive effect of all neighbours, in the orientation of each neighbour and scaled by the length of the shared interface. To extract the anisotropic part of the compression tensor we subtracted the isotropic part or trace of the compression tensor matrix. The resulting anisotropic tensor was negative in the principal axis of relative compression and positive in the orthogonal axis of relative tension (Fig. 3F).

#### Association between cell elongation and the anisotropic neighbour compression stress tensor

In Fig. 3G, we took the cell elongation log-ratios and long axis orientations and related these to the strength and orientation of neighbour tension (perpendicular to compression), as these should all be similar if there is a positive association between the measures. First, we normalised the log-ratios and neighbour tensions separately to their population standard deviations. Then we scaled the product of their normalised magnitudes by their co-orientation:

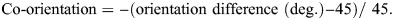


Association values ranged from above 1 (strongly positively correlated) to below −1 (strongly negatively correlated), zero if at 45° to each other or with zero magnitude in one or both measures.

## Supplementary Material

10.1242/develop.202862_sup1 Supplementary information
