# Supplementary material for: Mechanical stress combines with planar polarised patterning during metaphase to orient embryonic epithelial cell divisions
Source: Development. 2024 May 17;151(10):dev202862. doi: 10.1242/dev.202862 (PMC11165716; doi:10.1242/dev.202862)
Supplement: Supplementary information [file develop-151-202862-s1.pdf]

Figure 1 Supp

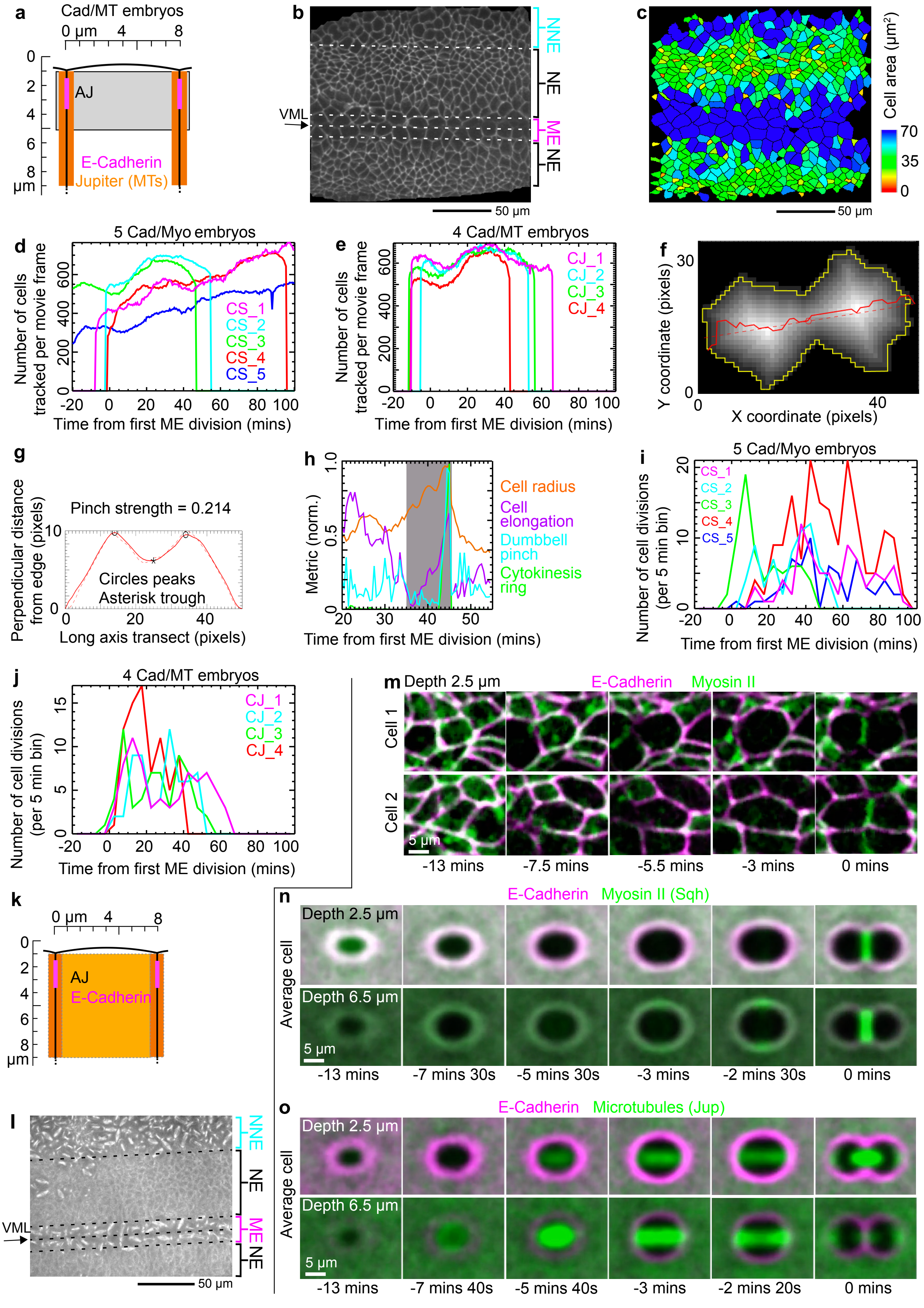

Development • Supplementary information

Fig. S1. Tracking cell divisions *in vivo*.
a) Schematic of epithelial cell apex showing the depth projection range (grey box) relative to cell apices used to track cells at the level of AJs in Cad/MT movies.
b) Projection of E-Cadherin channel at the level of AJs at 0 mins from Cad/MT movie CJ_3. Central horizontal band of large cells are mesectoderm (ME), straddling the ventral midline (VML). Neurectoderm (NE) cells flank the mesectoderm, with some lateral non-neural ectoderm (NNE) cells dividing at the top of the image. Anterior left.
c) Segmented cell shapes in (**b**), colour-coded by surface area.
d) Number of cells segmented and tracked successfully per image frame for five Cad/Myo movie. **e)** Same as **d)** for four Cad/MT movies.
f, g) Calculating 'dumbbell pinch' measure for an example cell outline (yellow). A mid-line path (red line) is calculated as the watershed of the distance to outline map (greyscale) along the long axis of the cell (**f**). This path is simplified to the regression line fit to its path points (dashed red line). The distance from pixel outline along this regression path has two peaks for a dumbbell shape (**g**). The difference in height between the lesser of the two peaks and the minimum between peaks, expressed as a proportion of the former, is used to quantify the strength of 'dumbbell pinch'.
h) Example classification of a cytokinesis event, here at 45 mins after the first mesectoderm division, using metrics color-coded as in Fig. 1f. Grey box shows the -10 to +0.5 mins window in which mitosis was identified by a high combinatorial score of an increasing cell radius over 10 mins prior to division, an increase in cell elongation over 2.5 mins prior to division, an increase in dumbbell pinch and cytokinesis ring in the last 1.5 mins, and an abrupt decrease in cell radius on division.
i) Frequency of cell division events over developmental time for five Cad/Myo embryo movies. 5 min bins. **j)** Same as **h)** for four Cad/MT embryo movies.
k) Schematic of epithelial cell profile showing the depth projection range (light orange box) relative to cell apices used to quantify cytoplasmic MT density in Cad/MT movies.
l) Projection of the depth range in **k)** from MT channel of Cad/MT movie CJ_4 at 10 mins. Lower horizontal band of mitotic spindles are mesectoderm (ME) cells straddling the VML (arrow). Top band is non-neural ectoderm (NNE). Some neurectoderm (NE) cells in between are starting to enter mitosis. Anterior right.
m) Cell fluorescence around cell centroids for two example Cad/Myo cells at the level of AJs, 2.5 µm from the surface of the epithelium.
n) Average cell fluorescence around tracked cell centroids from Cad/Myo embryos at depths 2.5 µm (top) and 6.5 µm (bottom) from the surface of the epithelium. Data pooled from 409 neurectoderm and NNE cells. See Supplementary Movie 3.
o) Average cell fluorescence around cell centroids from Cad/MT embryos at depths 2.5 µm (top) and 6.5 µm (bottom) from the surface of the epithelium. Data pooled from 274 neurectoderm and NNE cells. See Supplementary Movie 4.
In **m)**-**o)**, all cells and their surrounds have been rotated so that they divide horizontally.

Development • Supplementary information

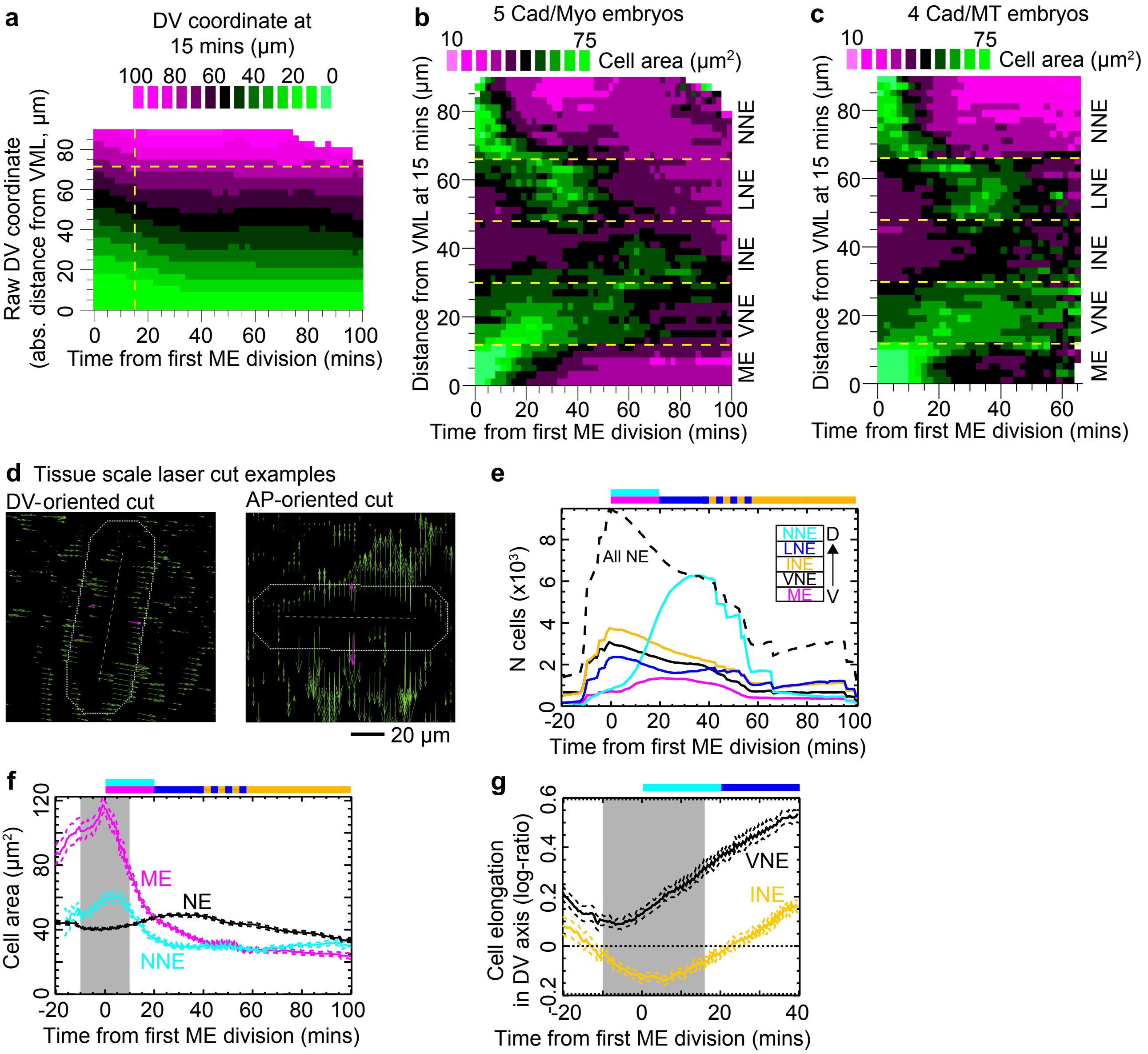

Fig. S2. Metaphase cell shape tracks tissue tension.

a) Flow of cells in DV across time showing that convergence stops in lateral tissue (dashed horizontal yellow line) at 15 mins after the first mesectoderm (ME) division (dashed vertical yellow line). Data pooled from five Cad/Myo embryos. See Fig. S1d for N cells.

b) Average cell areas for pooled data from five Cad/Myo embryos. Yellow dashed lines show manually assigned borders between DV domains with different patterns of cell shape. The DV y-axis is a co-moving coordinate frame assigned to cells at 15 mins (see **a)**). See Fig. S1d for N.

c) Average cell areas for pooled data from four Cad/MT embryos as in **b)**. See Fig. S1e for N.

d) AP (left) and DV (right) component of the PIV flow field in Sqh-GFP channel 2.9 sec after tissue scale laser cuts. Cut sites and orientation are indicated by the dashed line. The location of cuts is shown in the schematic in Fig. 2e.
e) N well-tracked cells over developmental time in each domain, pooled from five Cad/Myo and four Cad/MT embryos, used for plots Fig. 2f, i and **f)**, **g)**. Bin size 1 min.
f) Apical cell areas in mesectoderm and pooled neurectoderm mitotic domains over developmental time (see Fig. 2f for cell elongation). NNE cell areas are shown in Fig. 2i. Top bar as in Fig. 2b.
g) Cell elongation log-ratio in the DV axis for INE and VNE domains over developmental time. Top bar as in Fig. 2i.
Dashed lines either side of population means in **f)**, and **g)** are +/- 95% CIs.

Development • Supplementary information

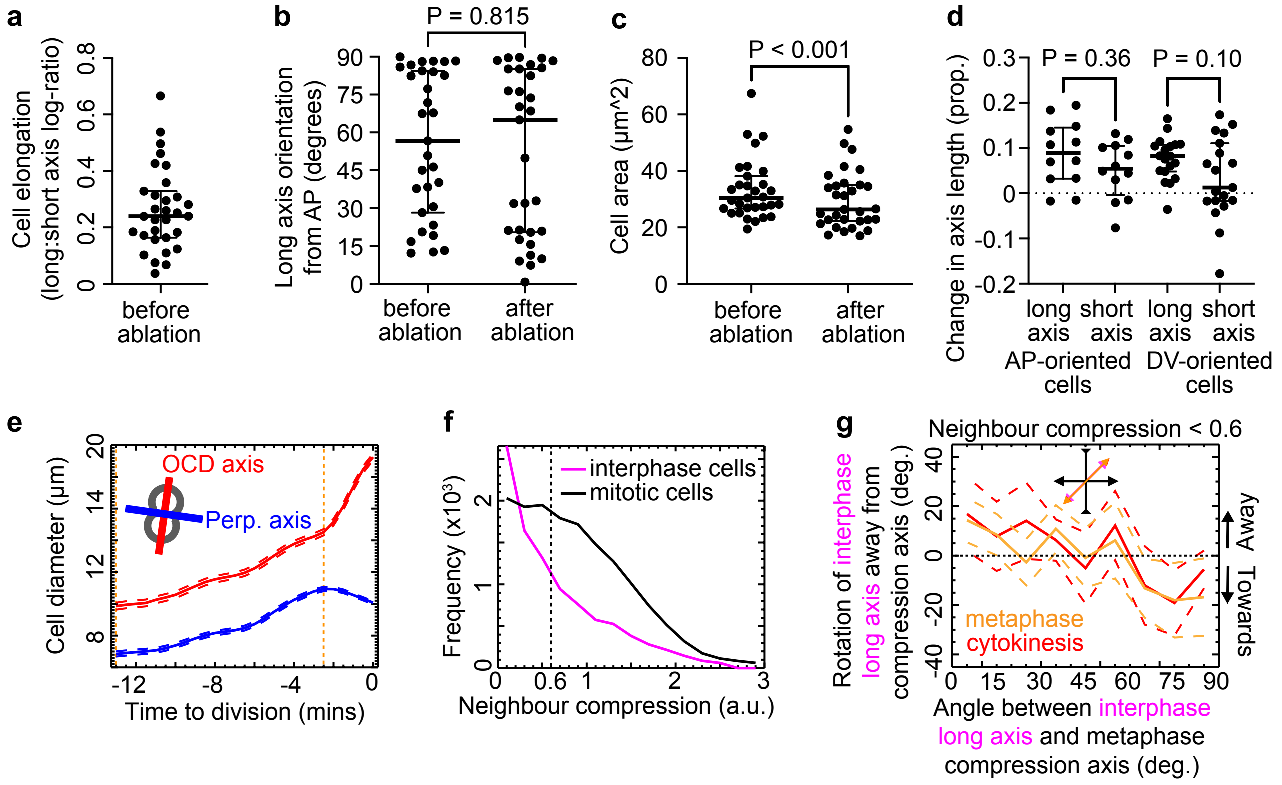

Fig. S3. Compression from neighbouring dividing cells during metaphase re-orients divisions
a-d) Box laser cut results.

a) Distribution of cell shape elongation before ablation of cells selected for box ablation (N = 31).
b) Distribution of the orientation of cell long axes before and 15 s after ablation, showing that a range of AP- and DV-oriented cells were selected, though it was difficult to find cells aligned perfectly with AP (Mann-Whitney test, N = 31).
c) Reduction in cell areas after box cut ablations (Paired t-test; N = 31).
d) Proportional reduction in axis lengths after box ablation, broken down by orientation of cell long axis before ablation (Paired t-tests; AP-oriented, N = 12; DV-oriented, N = 19).
e) Cell diameters in the future OCD (red) and orthogonal (blue) axes. All neurectoderm divisions from five Cad/Myo embryos, N = 336. Orange dotted vertical line marks the onset of anaphase.
f) Frequency of the strength of neighbour compression for all cells with at least one mitotic neighbour (within 13 minutes prior to cytokinesis). Because divisions occur in quasi-synchronous nests, neighbour compression is more frequent in mitotic than interphase cells. Vertical dotted line, threshold above which neighbour compression re-orients focal cell long axes (see Fig. 3g). Data pooled from five Cad/Myo and four Cad/MT embryos. Mitotic cells defined as within 13 minutes prior to division, interphase cells as less than 13 minutes before division. Bin size 0.2.

Development • Supplementary information

g) Fig. 3j repeated for cells that had dividing neighbours but where the strength of anisotropic neighbour compression in metaphase (-4 mins) was below the threshold at which cells changed shape (< 0.6, Fig. 3g). See explanatory schematic in Fig. 3i. Some rotation of the cell long axes away from the compression orientation is expected when these are aligned (x-axis = 0 deg) and some rotation towards the compression orientation is expected if these are already perpendicular (x-axis = 90 deg) from undirected dynamics. In between these angle limits, the metaphase long axis (orange) and OCD (red) are not different from the interphase long axis, unlike in cells with strong neighbour compression (> 0.6, Fig. 3j). Inset cartoon represents interphase long axes (magenta) at 45 deg remaining unchanged in metaphase (orange) and cytokinesis (red). Total N = 128 for divisions in all domains where anisotropic neighbour compression was weak (< 0.6) in metaphase (-4 mins). Data pooled from five Cad/Myo and four Cad/MT embryos.
Dashed lines straddling population means in **e)** and **g)** are +/- 95% CIs.

Development • Supplementary information

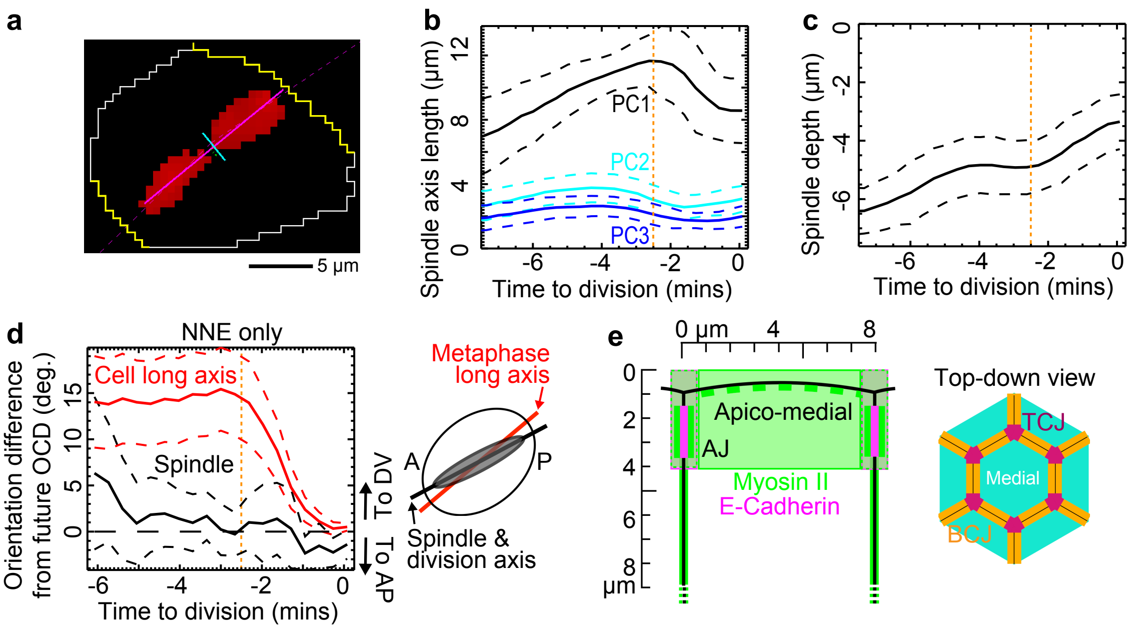

Fig. S4. An AP-oriented cue attracts the mitotic spindle independently of mechanics.

a) Example segmented cell outline (yellow) in late metaphase with pixels in MT channel above threshold (red) identified as spindle pixels. The orientation and dimensions of the spindle (red and blue lines) are calculated using 3D principal component analysis (PCA).
b) PCA of thresholded MT pixel volumes capture elongated shapes with one dominant axis. All except mesectoderm divisions pooled from four Cad/MT embryos, N divisions = 274**.**
c) Average depth of the spindle centre for the same data as in **b)**. Dashed lines straddling population means in **b)** and **c)** are +/- SD.
d) Angular difference, to AP or DV, between both the cell long axis and spindle and the OCD for non-neural ectoderm (NNE) cells. Dashed lines straddling population means are +/- 95% CIs. N divisions = 105.
e) Schematic of the apical depth range used to quantify Myosin-II at AJs and apico-medially. Top-down view shows how cell pixels are assigned to one of bi-cellular junctions (BCJs), tri-cellular junctions (TCJs), and apico-medial (see Methods).
Orange line in **b-d)** marks the start of anaphase.

Development • Supplementary information

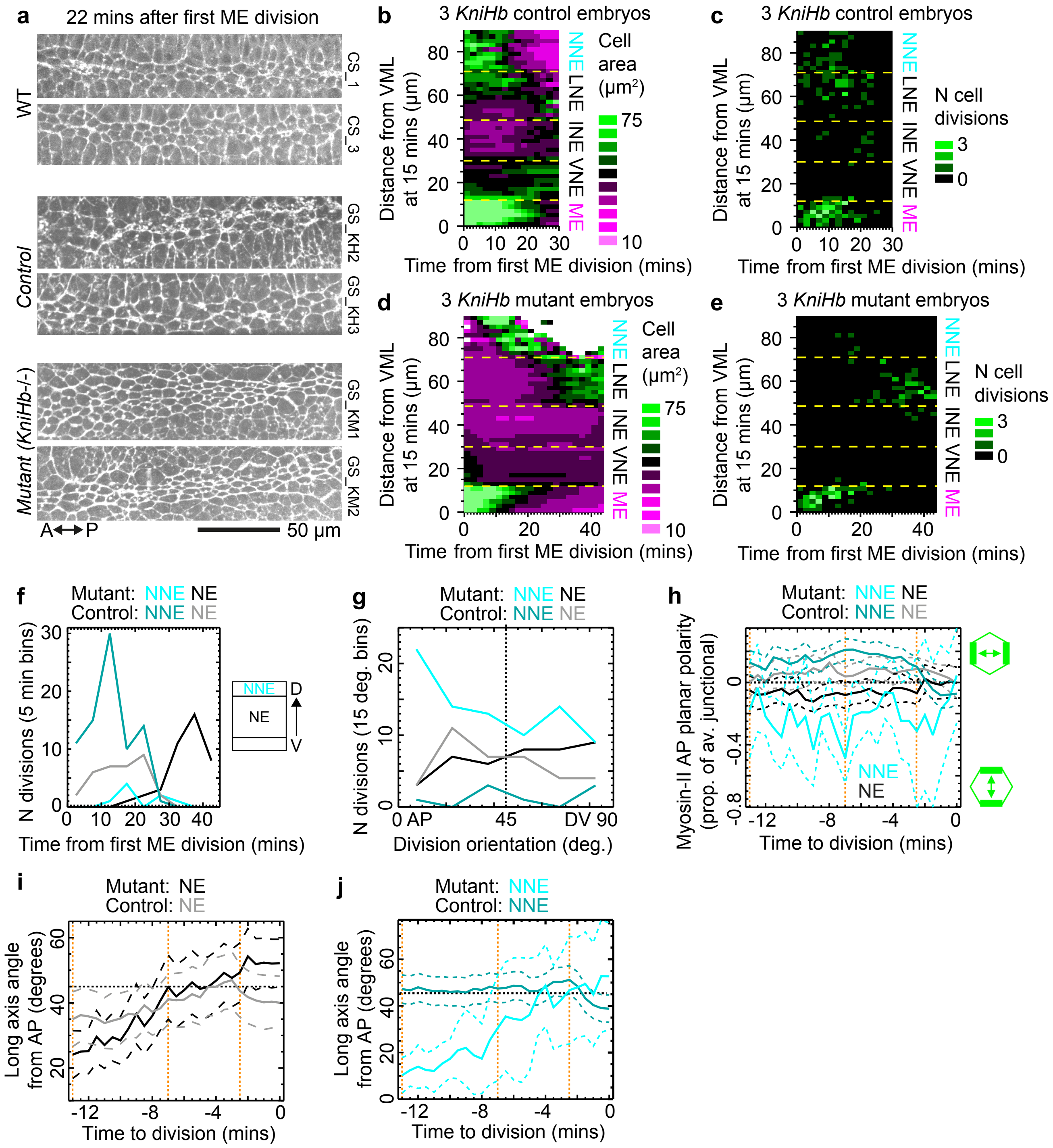

Fig. S5. Anaphase long cell axis rotation towards AP is lost in an AP-patterning mutant

a) Stills from E-Cadherin channel from two further example embryos per genotype (see Fig. 5a) showing the width of the mesectoderm after division.

b-c) Data pooled from three *KniHb* control embryos.

b) Patterns of apical cell area over developmental time and across DV (compare with Fig. 2Sb, c).

c) Patterns of cytokinesis events over developmental time and across DV (compare with Fig. 2b).

d-e) As **b-c)** but for data pooled from three *KniHb-/-* mutant embryos.

f-j) Data pooled from three KniHb mutant embryos are compared to three KniHb control embryos. N divisions: Mutant non-neural ectoderm (NNE), 8; mutant neurectoderm (NE), 41; control NNE, 81; control NE, 33.

Development • Supplementary information

f) Frequency of divisions over developmental time.

g) Frequency of division orientation.

h) Average Myosin-II planar polarity through mitosis by mitotic domain. Mutant data is negative on average, indicating DV-oriented planar polarity, compared to AP-oriented planar polarity in controls. Vertical orange lines in **h-j)** mark the effective onset of prophase (-13 mins), metaphase (-7 mins) and anaphase (-2.5 mins).

i) Angle of cell long axis from AP during mitosis for neurectoderm cells, showing the loss of anaphase rotation to AP in the mutant.

j) As in **i)** but for NNE cells.

Development • Supplementary information

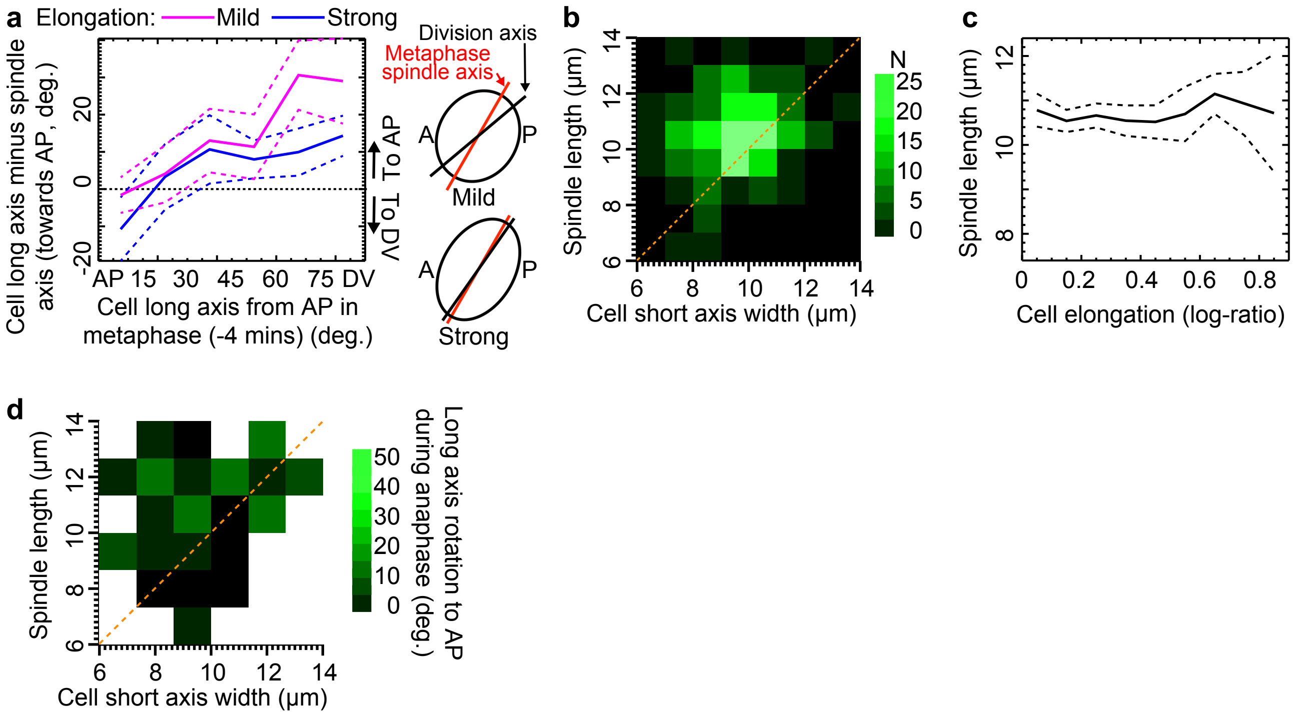

Fig. S6. Interaction between mechanics and patterning

a) The metaphase (-4 mins) spindle is consistently towards AP of the cell long axis on average, more strongly in less elongated cells (log-ratio < 0.3). Cells with long axes already oriented towards AP in metaphase cannot have a spindle more towards AP (left) while the spindle of DV-oriented cells has scope to be aligned more towards AP. All data from four Cad/MT embryos are shown (N divisions: Mild = 157, strong = 158).

b) By cell dependence of spindle length on cell short axis width in metaphase (-4 mins). Spindles exceed short axis widths in most cells (above and to the left of the dashed orange line, y=x). All divisions from four Cad/MT embryos are shown (N = 315).

c) Spindle length is not correlated with cell elongation in metaphase (-4 mins). All divisions from four Cad/MT embryos are shown (N = 315).

d) There is no consistent pattern to ‘anaphase rotation’ on a plot of spindle length versus short axis length, for cells oriented towards AP (< 45 deg.) in metaphase (-4 mins). Orange dotted line, y=x. See **b)** for N.

Dashed lines straddling population means in all line-graphs are +/- 95% CIs.

Development • Supplementary information

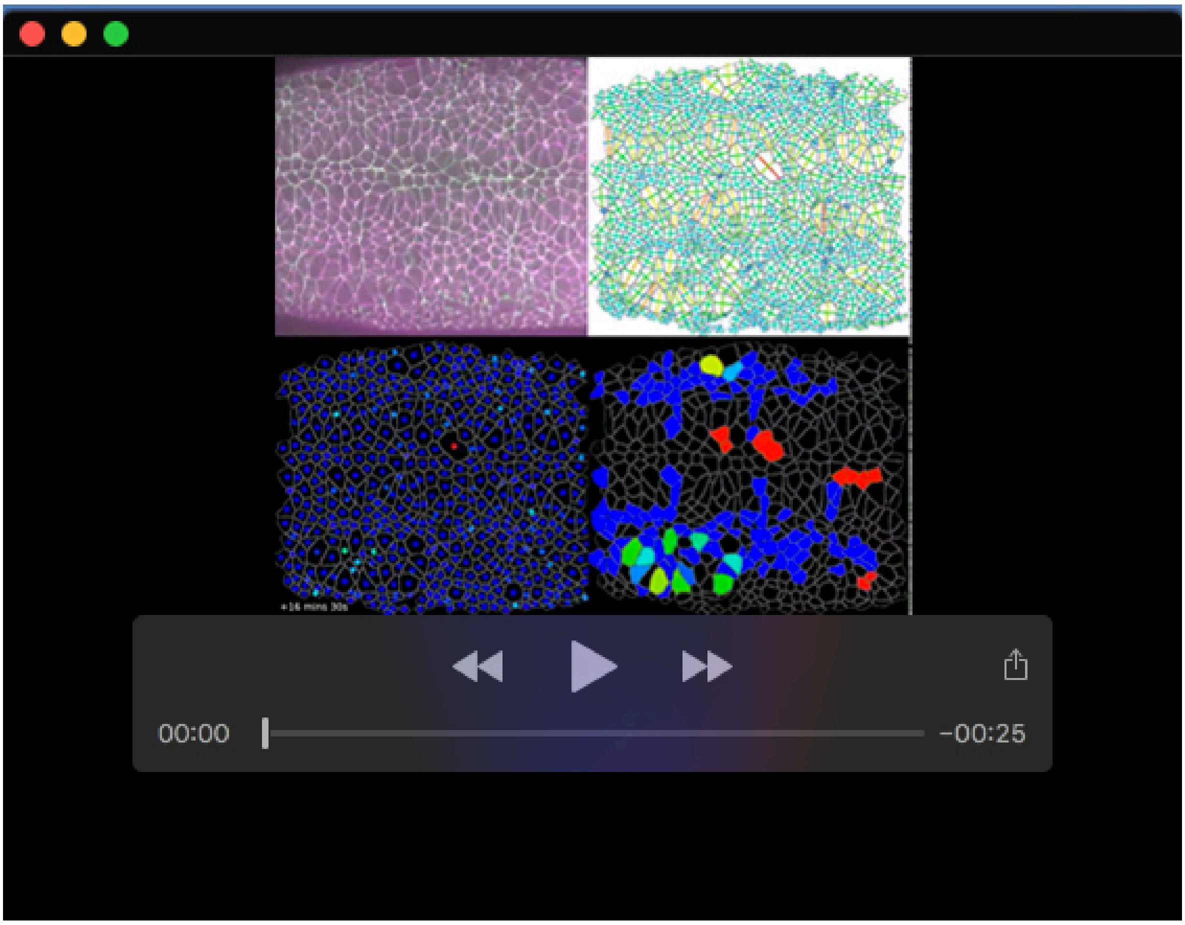

Movie 1. Classifying cell division events in raw movies. Top left; E-Cadherin (magenta) and Myosin-II (green) fluorescence confocal movie channels. Top right; best-fit ellipses drawn on top of segmented and tracked cell shapes. Lengths of principal axes are colour-coded blue (1 µm) to green (5.5 µm) to red (10 µm). Bottom left; dumbbell pinch measure for identifying cytokinesis, increasing from zero (blue) to 0.2 (red). Bottom right; final classification of cytokinesis events. Cells are colour-coded by time to cytokinesis from blue (<= -10 mins) to red (>= 0 mins). Images and data from Cad/Myo embryo CS_4.

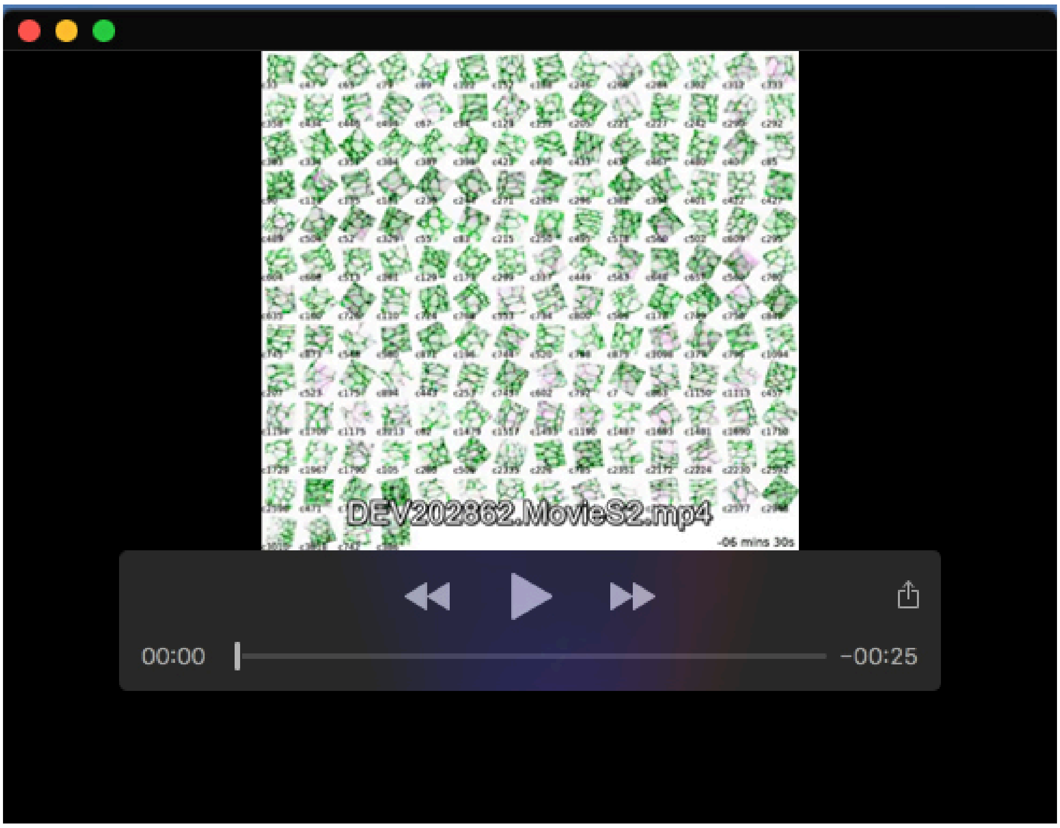

Movie 2. Classified cell division events. All 172 cell divisions classified in Cad/Myo movie CS_4 are portrayed in 30 s frame intervals from -11 mins to 0 mins, the frame before the mother cell splits into two daughters. The mother cell centroid is set to the centre of each square neighbourhood, which has been rotated so that the orientation of division is horizontal (as in Fig. 1e and in Fig. 1S2). Magenta is Shotgun-GFP (E-Cadherin) and green is Sqh-mCherry (Myosin-II). Cytokinesis rings can be seen appearing as vertical green lines in the centre of dividing cells at around -2.5 mins.

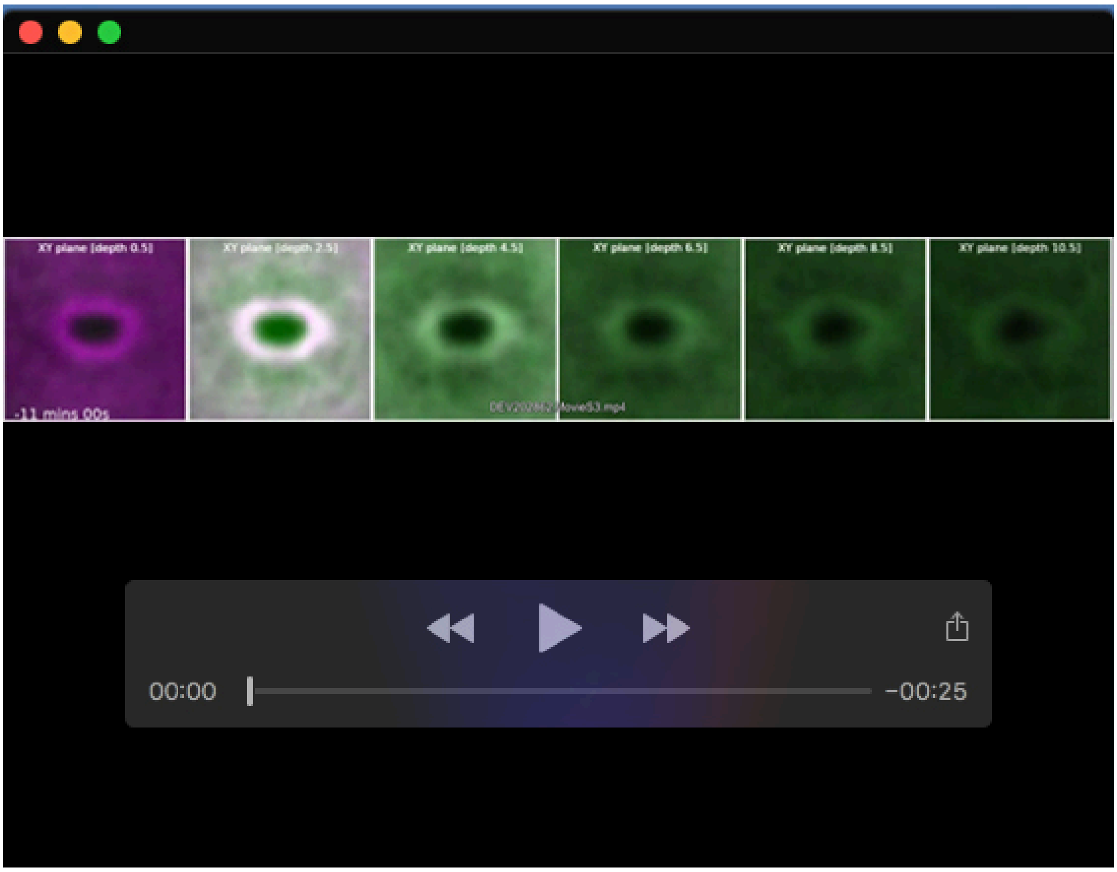

Movie 3. Average Cad/Myo mitosis behaviour. Average cell fluorescence through mitosis from -13 mins to cytokinesis (0 mins), at 30 s frame intervals. Left panel is at depth 0.5 µm below the surface of the epithelium with subsequent panels stepping 2 µm deeper to 10.5 µm in right panel. Magenta is E-Cadherin, green is Myosin-II. Scale bar as in Fig. 1S2b.

Development • Supplementary information

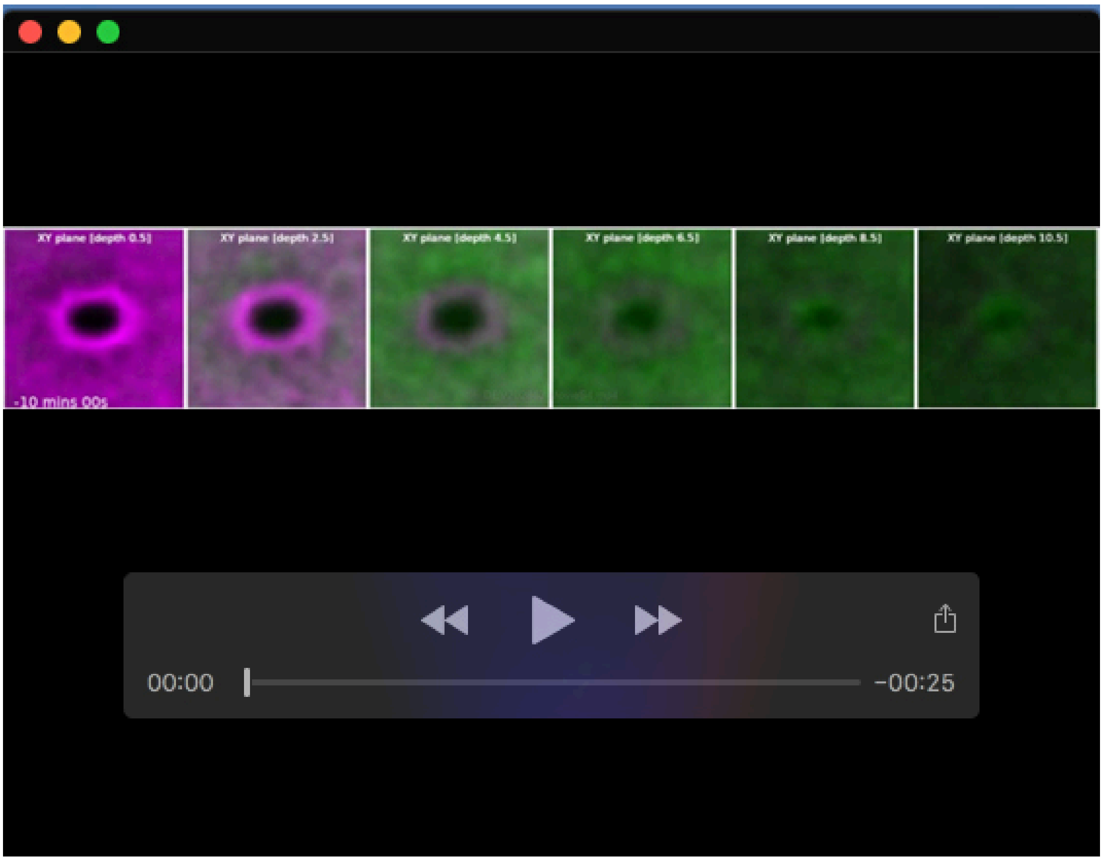

Movie 4. Average Cad/MT mitosis behaviour. Average cell fluorescence through mitosis from -13 mins to cytokinesis (0 mins), at 20 s frame intervals. Left panel is at depth 0.5 µm below the surface of the epithelium with subsequent panels stepping 2 µm deeper to 10.5 µm in right panel. Magenta is E-Cadherin, green is MTs. Scale bar as in Fig. 3d.

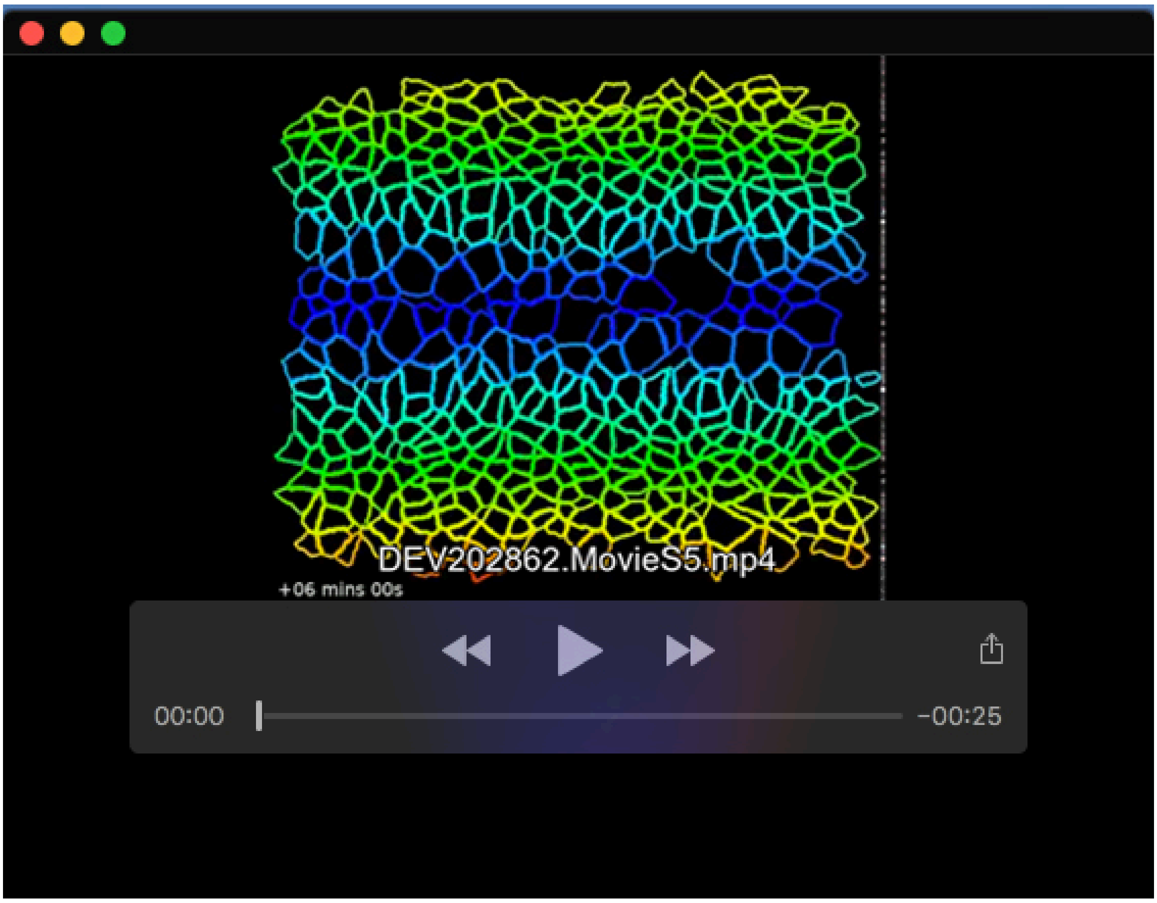

Movie 5. Embryo DV coordinates. Example embryo axial coordinates for movie CS_4. Colour-coded DV distance from ventral mid-line at 15 mins, carried forwards and backwards by cells as a co-moving coordinate frame. Blue is 0 µm, red is 84 µm from VML.

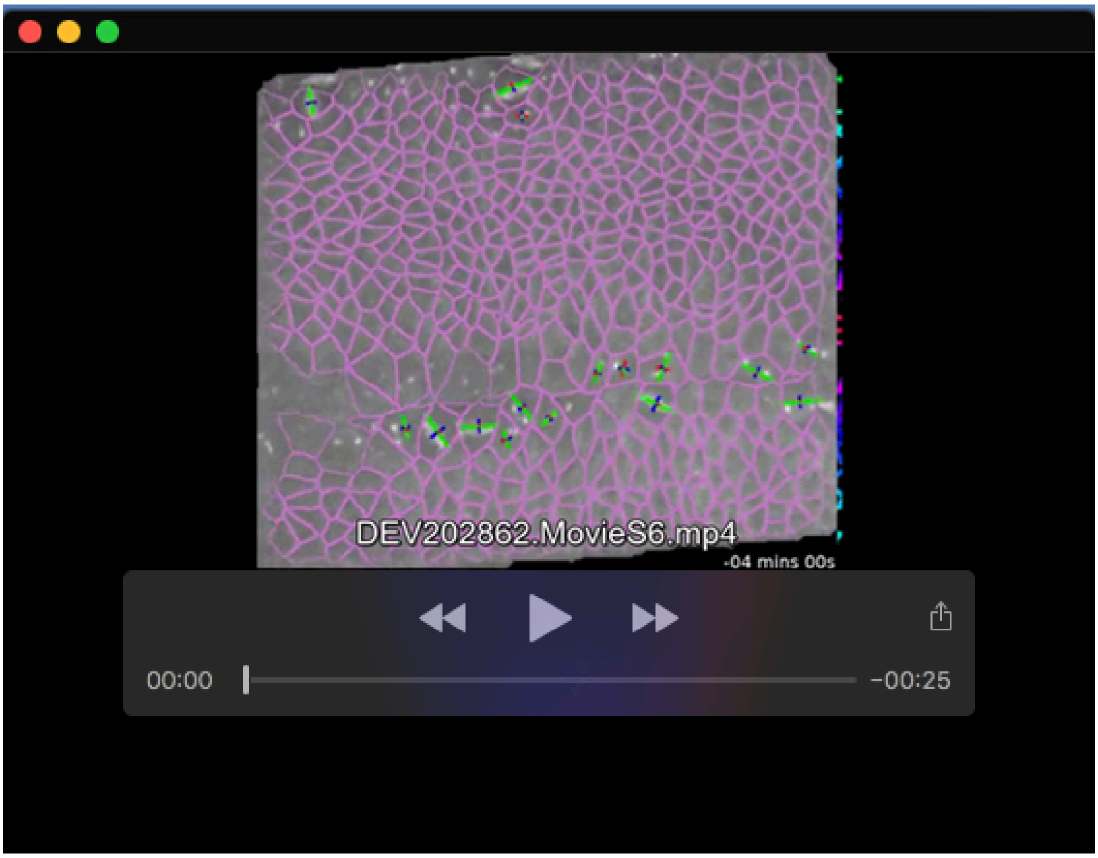

Movie 6. Jupiter spindle tracking. 3D ellipsoid spindle shapes were extracted from volume images of intra-cellular (cytoplasmic) Jupiter fluorescence. Principal axes of spindle shape are drawn on each cell's centroid, longest first in the colour sequence green, red and blue. Tracked cell shapes are in pink. Embryo move CJ_4 is shown.

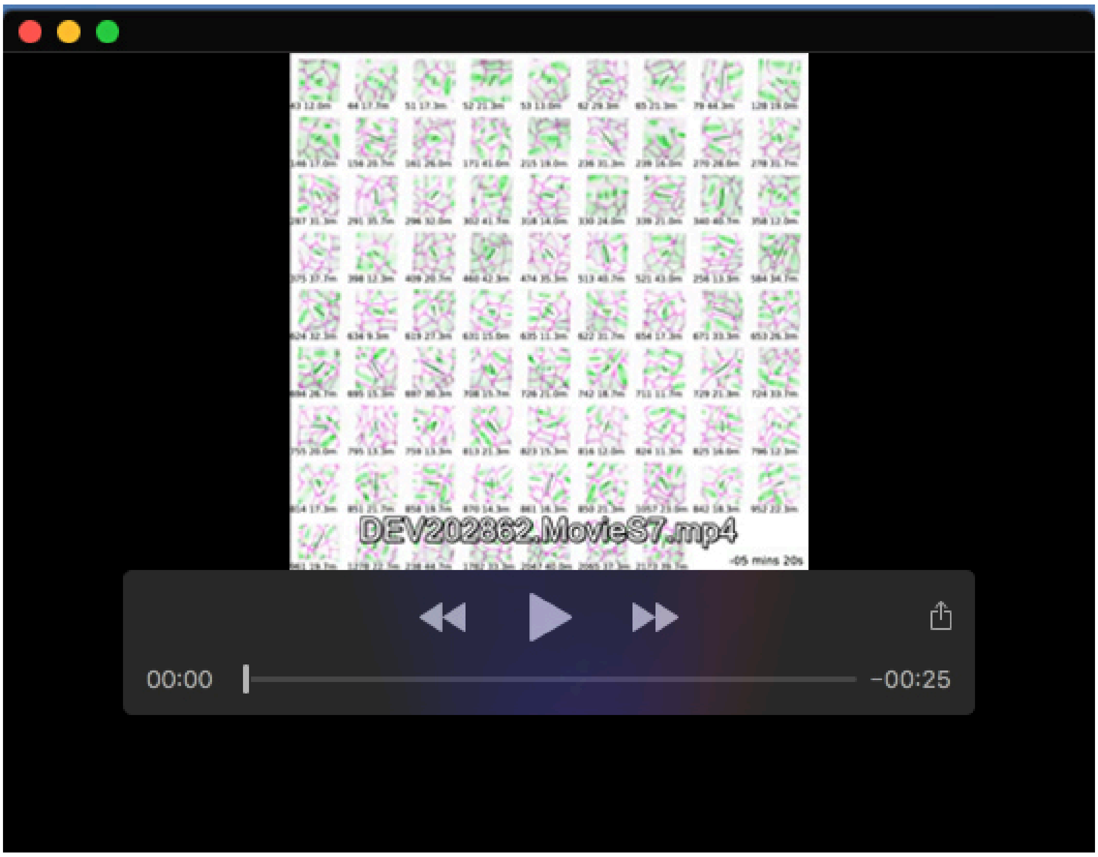

Movie 7. Spindle versus cell long axis orientations. All 79 cell divisions classified in Cad/MT movie CJ_4 are portrayed in 20 s frame intervals from -11.7 mins to 0 mins, the frame before the mother cell splits into two daughters. The mother cell centroid is set to the centre of each square neighbourhood, which has been rotated so that the embryonic AP axis is horizontal (unlike Supplementary Movie 2). Magenta is E-Cadherin and green is Jupiter (MTs). Mitotic spindles can be seen appearing after NEBD at around -7 mins and elongating during anaphase from around -2.5 mins. Overlaid on each focal cell centroid is a black line representing the orientation and strength of elongation (see Fig. 4e, f for scale bars). Though there are exceptions, on average spindles are towards AP (horizontal) of cell long axes.
